# Prediction of the infecting organism in peritoneal dialysis patients with acute peritonitis using interpretable Tsetlin Machines

**DOI:** 10.1093/bioadv/vbaf140

**Published:** 2025-06-19

**Authors:** Olga Tarasyuk, Anatoliy Gorbenko, Matthias Eberl, Nicholas Topley, Jingjing Zhang, Rishad Shafik, Alex Yakovlev

**Affiliations:** School of Engineering, Newcastle University, Newcastle upon Tyne, NE1 7RU, United Kingdom; School of Built Environment, Engineering and Computing, Leeds Beckett University, Leeds, LS1 3HE, United Kingdom; Division of Infection & Immunity, School of Medicine, Cardiff University, Cardiff, CF14 4XN, United Kingdom; Systems Immunity Research Institute, Cardiff University, Cardiff, CF14 4XN, United Kingdom; Division of Infection & Immunity, School of Medicine, Cardiff University, Cardiff, CF14 4XN, United Kingdom; Systems Immunity Research Institute, Cardiff University, Cardiff, CF14 4XN, United Kingdom; School of Engineering, Newcastle University, Newcastle upon Tyne, NE1 7RU, United Kingdom; Division of Infection & Immunity, School of Medicine, Cardiff University, Cardiff, CF14 4XN, United Kingdom; Systems Immunity Research Institute, Cardiff University, Cardiff, CF14 4XN, United Kingdom; School of Engineering, Newcastle University, Newcastle upon Tyne, NE1 7RU, United Kingdom; School of Engineering, Newcastle University, Newcastle upon Tyne, NE1 7RU, United Kingdom

## Abstract

**Motivation:**

The analysis of complex biomedical datasets is becoming central to understanding disease mechanisms, aiding risk stratification and guiding patient management. However, the utility of computational methods is often constrained by their lack of interpretability, which is particularly relevant in clinically critical areas where rapid initiation of targeted therapies is key.

**Results:**

To define diagnostically relevant immune signatures in peritoneal dialysis patients presenting with acute peritonitis, we analysed a comprehensive array of cellular and soluble parameters in cloudy peritoneal effluents. Utilizing Tsetlin Machines, a logic-based machine learning approach, we identified pathogen-specific immune fingerprints for different bacterial groups, each characterized by unique biomarker combinations. Unlike traditional ‘black box’ machine learning models, Tsetlin Machines identified clear, logical rules in the dataset that pointed towards distinctly nuanced immune responses to different types of bacterial infection. Importantly, these immune signatures could be easily visualized to facilitate their interpretation, thereby allowing for rapid, accurate and transparent decision-making. This unique diagnostic capacity of Tsetlin Machines could help deliver early patient risk stratification and support informed treatment choices in advance of conventional microbiological culture results, thus guiding antibiotic stewardship and contributing to improved patient outcomes.

**Availability and implementation:**

All underlying tools and the anonymized data underpinning this publication are available at https://github.com/anatoliy-gorbenko/biomarkers-visualization.

## 1 Introduction

Reliable, rapid and accurate diagnosis of infection remains an unmet clinical need. Microbiological culture can take several days to generate results and is often impacted by inadequate sample quality, contamination and problems with fastidious or slow-growing organisms (Chakera *et al.* 2018). Molecular techniques to detect pathogens such as mass spectrometry or polymerase chain reaction equally depend on sample quality, may yield negative results at low pathogen numbers and do not discriminate between live and dead organisms. Finally, host biomarkers in patients presenting with suspected infections are often relatively unspecific due to the highly dynamic and individual nature of the early immune response. Despite promising advances, no single biomarker is sufficiently specific or sensitive to accurately predict the presence of an infection or indeed the type or even species of causative pathogen (Chakera *et al.* 2018). Factors such as patient age and gender, comorbidities, infection severity, pathogen type and virulence can all influence biomarker expression patterns, as can medication. This creates a challenge for the accurate identification of robust immune signatures that could guide more rapid diagnosis and targeted antibiotic treatment (Aufricht *et al.* 2017). The complexity of biomarker profiles necessitates sophisticated multi-parameter analysis techniques, leading to an increased interest in applying machine learning (ML) methods to biomedical datasets, aiming to improve patient stratification and tailor therapies more effectively.

ML models such as support vector machines (SVMs), artificial neural networks (ANNs) and random forests (RFs) have been successfully applied to biomedical datasets (Peiffer-Smadja *et al.* 2020, Ahsan *et al.* 2022), including those from our own work in patients with urinary tract infection (Gadalla *et al.* 2019), peritoneal dialysis (PD)-related peritonitis (Zhang *et al.* 2017) and sepsis (Burton *et al.* 2024). However, the lack of explainability in most ML models, i.e. the lack of understanding and hence of confidence in what they show, is a major barrier to their wider clinical adoption. Many ML methods, especially intricate ones like ANNs, often function as ‘black boxes’ (Rudin 2019), making it difficult for scientists, healthcare professionals and regulatory authorities to understand and trust the basis of ML-based predictions.

New ML techniques that are better interpretable are being developed to bridge this gap, such as approaches using decision trees (Mienye and Jere 2024), probabilistic and fuzzy logic (Zheng *et al.* 2024), or focusing on explainable artificial intelligence tools and post-hoc interpretability methods like SHapley Additive exPlanations (SHAP) (Burton *et al.* 2024, Salih *et al.* 2025). However, their computational demands, difficulty of standardization and challenges in usability continue to hinder their widespread adoption. Indeed, current explainable artificial intelligence tools often provide explanations in technical or abstract terms that may not align with the clinical reasoning process. The creation of user-friendly interfaces and clinical decision-support systems that integrate seamlessly with existing workflows remains an area of active research.

This paper aims at providing explainability in decision-making via employing a relatively new logic-based ML algorithm called Tsetlin Machine (TM), for which explainability is an intrinsic feature. TMs rely on the collective behaviour of learning automata (Tsetlin 1973, Varshavsky and Pospelov 1988, Narendra and Thathachar 2012). As part of their training and inference, TMs generate a set of conjunctive logical statements (logical clauses that can be viewed as directly interpretable inference rules), which vote for or against each class, thus justifying the decision-making. TMs have considerably fewer hyperparameters to tune than other ML methods. These are highly interpretable since their model prediction is carried out via proposition logic clauses. The logical rules can be naturally visualized, which further eases their understanding and interpretation by specialists. The fact that TMs use Boolean (i.e. semi-quantitative) features as input data, unlike other ML methods that operate with continuous numerical values, makes them particularly attractive for deciphering biological processes. As a consequence, for clinical use, they may allow easier translation of complex datasets generated using laborious techniques into the design of simpler methods such as lateral flow tests, while maintaining competitive accuracy.

We describe the application of TM-based analysis techniques to a set of soluble and cellular biomarkers measured previously in individuals presenting with acute PD-related peritonitis (Zhang *et al.* 2017). PD is a life-saving renal replacement therapy used to manage end-stage kidney disease by removing waste products, excess fluid and toxins from the body, utilizing the peritoneal membrane lining the abdominal cavity as a semipermeable filter. Despite its effectiveness and convenience for many patients, PD carries the risk of peritonitis, a severe infection of the peritoneal cavity (Li *et al.* 2022). Peritonitis is a significant complication that can arise from contamination during catheter handling, bowel leakage and other reasons, and is associated with significant morbidity, treatment failure and in some cases death (Cho *et al.* 2024). Moreover, any inflammatory episode of peritonitis may cause scarring and thickening of the peritoneal membrane (Fielding *et al.* 2014) and potentially contribute to treatment failure. Timely diagnosis and antimicrobial intervention are thus key to successful treatment (Chakera *et al.* 2018). However, despite the recognition more than three decades ago that levels of inflammatory markers are elevated in the peritoneal effluent hours prior to the manifestation of overt clinical symptoms (Betjes *et al.* 1996), a simple biomarker-based lateral flow test for early peritonitis was only recently developed (Goodlad *et al.* 2020, Htay *et al.* 2024) and has not been widely adopted.

It is well recognized that different classes (Gram-positive and Gram-negative bacteria) and species of micro-organisms result in different patient outcomes and that infections with them give rise to distinct sets of biomarkers (‘immune fingerprints’) (Liuzzi *et al.* 2016, Zhang *et al.* 2017). Immune fingerprints have shown promise for rapid point of care prediction of infection (Gadalla *et al.* 2019) and causative pathogen (Zhang *et al.* 2017, Burton *et al.* 2024) in other infectious contexts, potentially allowing early risk stratification and targeted antibiotic treatment. By combining biomarker measurements during acute peritonitis and logic-based inference approaches as offered by TMs, we now demonstrate the power of interpretable ML models to analyse complex biomarker signatures and guide decision-making processes. Our findings may have immediate diagnostic implications, potentially guiding appropriate antibiotic treatment before conventional microbiological culture results become available.

## 2 Methods

### 2.1 Patients and biomarker dataset

The study cohort comprised 82 adults receiving peritoneal dialysis (PD) who were admitted between 2008 and 2016 to the University Hospital of Wales in Cardiff (UK) with acute peritonitis. Clinical diagnosis of peritonitis was based on the presence of abdominal pain and cloudy peritoneal effluent with >100 white blood cells per mm^3^ (Li *et al.* 2022). According to the microbiological analysis of the effluent, peritonitis episodes were defined as culture-negative or as confirmed bacterial infections by a Gram-positive or Gram-negative organism (Supplementary Tables S1 and S2, available as supplementary data at *Bioinformatics Advances* online). Cases of fungal infection and mixed or unclear culture results were excluded. The study was approved by the South East Wales Local Ethics Committee (04WSE04/27) and registered on the UK Clinical Research Network Study Portfolio under reference number #11838 ‘Patient immune responses to infection in peritoneal dialysis’ (PERIT-PD). All individuals provided written informed consent.

To identify organism-specific immune fingerprints and predict the causative pathogen, a wide range of 9 cellular and 40 soluble immune biomarkers was measured in the cloudy effluent of all 82 PD patients presenting with acute peritonitis (Supplementary Tables S1 and S2, available as supplementary data at *Bioinformatics Advances* online). These biomarkers included frequencies and total numbers of infiltrating leukocyte populations as well as levels of inflammatory mediators and tissue damage-associated molecules, covering the breadth and complexity of the local immune response to infection (Zhang *et al.* 2017) (Supplementary Table S3, available as supplementary data at *Bioinformatics Advances* online).

### 2.2 Machine learning with interpretable Tsetlin Machine

TMs leverage the collective behaviour of learning automata and bases its inference on interpretable logic-based rules, specifically conjunctive clauses (Granmo 2018, Lei *et al.* 2020). These clauses logically link together input features, thereby creating distinct patterns that represent different classes, enabling TMs to make transparent and interpretable predictions of infecting organisms in peritoneal dialysis patients with acute peritonitis. TMs are an actively evolving field of research where novel architectures and training methods are being developed and becoming available via the GitHub repository (Centre for Artificial Intelligence Research 2018). Here, the Python implementation of the basic *MulticlassTsetlinMachine* from the *pyTsetlinMachineParallel* package (v.0.2.1) was used. The source code is available at https://github.com/cair/pyTsetlinMachineParallel.

A TM has three major hyperparameters affecting its performance and defining a balance between clauses generalization and specialization, namely: the number of clauses per class *C* (i.e. the number of logical rules used for inference), the voting threshold *T* and the learning sensitivity *s* that regulates the trade-off between clauses generalization and specialization. When data samples of different classes share a higher degree of similarity, a higher value of *s* enables the TM to effectively distinguish subtle class-specific patterns. During the tuning of TM hyperparameters, the number of clauses *C* was set to 20 in the present study, with 10 *positive* clauses creating class patterns and 10 *negative* clauses generating patterns for the counter-class(es). This provided a reasonable balance between achieving high classification accuracy and maintaining ease of comprehension and interpretation of the resulting logical rules. The voting threshold *T* and the learning sensitivity *s* were set to their optimum values as described (Tarasyuk *et al.* 2023). In particular, the global optimum of the voting threshold *T* for the given number of clauses *C* approximates to the square root of *C*/2, which maximizes voting power of each clause according to Jagiellonian compromise for qualified majority in the Penrose’s square root voting system (Penrose 1946). When data samples of different classes share a higher degree of similarity, a higher value of *s* enables the TM to effectively distinguish subtle class-specific patterns.

Tsetlin automata (Narendra and Thathachar 2012) in the TM serve as fundamental units for decision-making and learning, similar to artificial neurons in ANNs, although their roles and mechanisms differ significantly (Lei *et al.* 2020, Tarasyuk *et al.* 2023a). The ANNs used in our previous study (Zhang *et al.* 2017) were adopted here as reference ML model, configured with 40 000 neurons in the hidden layer for multi-class classification and 13 000 neurons for binary classification. This setup ensured comparable complexity to TMs configured with 20 clauses per class, utilizing up to 39 200 and 12 800 Tsetlin automata at maximum configuration, respectively.

### 2.3 Data preprocessing

#### 2.3.1 Data imputation

The original dataset had some missing biomarker values due to incomplete or failed measurements (Supplementary Table S4, available as supplementary data at *Bioinformatics Advances* online) (Zhang *et al.* 2017). Missing data were imputed to fit gaps by adopting Multivariate Imputation by Chained Equations (MICE) implemented in R package *mice* (v.3.17.0), which imputes an incomplete feature by generating synthetic values considering their relationship with other biomarkers (van Buuren and Groothuis-Oudshoorn 2011). Only one imputation was conducted by using RF for each individual column. The random seed was set to 500 to ensure reproducibility of results, and the maximum number of iterations for the chained equations algorithm was set to 50 in refining the imputed values.

#### 2.3.2 Data Booleanization

TMs operate on Boolean input data where input variables should be transformed into a set of Boolean literals, each signalling whether the value is within or outside a certain range. TMs naturally thus align with laboratory techniques such as lateral flow tests (LFTs) that present results in binary or low-level semi-quantitative bands. In contrast, continuous values such as biomarker levels determined by ELISA as used in this study must first be discretized into bins (quartiles or semi-quantitative intervals) that reflect clinically meaningful ranges like ‘baseline’, ‘moderate’, ‘elevated’, and ‘hyperinflammatory’. Such Booleanization can be viewed as a kind of data pre-processing similar to normalization procedures required for other ML algorithms.

Booleanization simplifies datasets by reducing feature granularity, making it easier to analyse and interpret in certain applications, such as rule-based ML or visualization. Too few bins, however, may blur fine biochemical dynamics of immune responses and affect classification accuracy, while too many bins expand the Boolean feature set, so that the TM must allocate more Tsetlin Automata, increasing memory footprint, used computational resources and training time. Our experiments with different numbers of semi-quantitative ranges illustrate this three-way trade-off: coarser Booleanization improves interpretability and resource efficiency, whereas finer binning demands greater computational resources, but may yield higher predictive performance.

To Booleanize the input dataset and convert biomarker values from quantitative to semi-quantitative Boolean features we used a simple binning method. For each biomarker we determined its range as the difference between the measured maximum and minimum values, which was then divided into equal intervals (bins). These intervals were then encoded using ‘one-hot encoding’ method, so that each biomarker is represented by a unique binary vector with the same length as the number of intervals. In this vector, only one element is set to 1 (*True*), indicating the presence of the biomarker value in that specific interval, and all other elements are set to 0 (*False*). For instance, using four semi-quantitative intervals per value, each of the 82 patient samples comprising 49 biomarkers was represented by 196 Boolean features (four bits/Boolean features per biomarker) (Supplementary Fig. S1, available as supplementary data at *Bioinformatics Advances* online).

#### 2.3.3 Data balancing and model cross-validation

**Figure 1.**
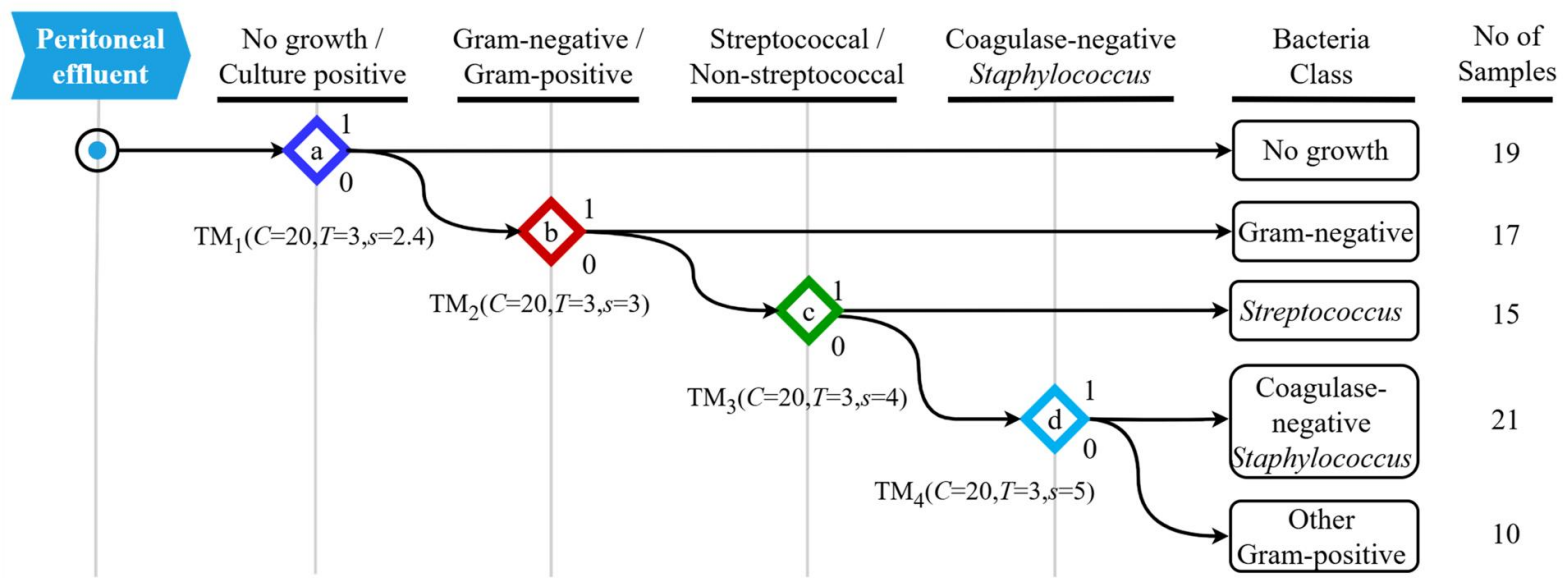
Hierarchical classification methodology to identify local immune fingerprints associated with peritonitis caused by different types of bacteria. A binary decision tree attempts to predict the causative organism in the following order: (a) discrimination between episodes of peritonitis that yielded no microbiological growth versus culture-positive episodes; (b) discrimination between episodes caused by Gram-negative bacteria within the culture-positive group of patients; (c) discrimination between episodes caused by streptococcal organisms versus episodes caused by non-streptococcal Gram-positive bacteria; and (d) discrimination between episodes caused by coagulase-negative *Staphylococcus* versus episodes caused by other Gram-positive bacteria. The TM learning sensitivity *s* was optimized individually at each step for maximum performance.

The peritonitis cohort contained an unequal distribution of patients across different classes (Fig. 1), which resulted in an unbalanced dataset. To avoid biasing ML models toward the majority class(es) and poor performance on the minority class(es) and to ensure that TM learning automata of different classes receive equal reinforcements, we evaluated classification performance of the TMs and reference ANN models using stratified five-fold cross-validation, so that each fold preserved the original class proportions. Within each fold, data were partitioned into a training (80%) and a validation set (20%) combined with oversampling inside each training fold using *RandomOverSampler* from *imblearn* Python package (v.0.0). This stratified split allowed relative class frequences to be preserved in training and validation subsets. Random over-sampling was applied to the minority classes by selecting samples at random with replacement, thus keeping the probability of selecting any specific sample constant. This approach ensured more reliable performance estimates and provided a balanced training dataset within each fold, facilitating equal reinforcement opportunities across classes.

## 3 Results

### 3.1 Simultaneous discrimination through multiclass classification

To identify organism-specific immune fingerprints and predict the causative pathogen, a wide range of cellular and soluble immune biomarkers was measured in the cloudy effluent of PD patients presenting with acute peritonitis (Supplementary Tables S1 and S2, available as supplementary data at *Bioinformatics Advances* online). These biomarkers included frequencies and total numbers of infiltrating leukocyte populations as well as levels of inflammatory mediators and tissue damage-associated molecules, covering the breadth and complexity of the local immune response to infection (Zhang *et al.* 2017) (Supplementary Table S3, available as supplementary data at *Bioinformatics Advances* online).

An earlier attempt to define immune fingerprints that would simultaneously discriminate patients with all major groups of infecting organisms showed relatively low validation performance for different ML techniques such as SVMs, RFs and ANNs, despite high training accuracy (Zhang *et al.* 2017). Reassuringly, TMs demonstrated considerable improvement in multiclass classification and allowed to reach 59.63 ± 8.82% of validation accuracy on average across five folds while maintaining 98.59 ± 1.88% train accuracy on the whole dataset (Table 1).

**Table 1.** Multi-class classification train and validation accuracy of TM.^a^

Dataset	Data split	TM (*C *= 20, *T *= 3, *s *= 3) performance (%)			
		Accuracy	Precision	Recall	F1 Score
Full dataset (40 soluble, 9 cellular biomarkers)	Train	98.59 ± 1.88	98.74 ± 1.68	98.59 ± 1.88	98.55 ± 1.95
	Validation	59.63 ± 8.82	54.87 ± 9.99	56.40 ± 9.43	52.71 ± 8.80
Soluble biomarkers (excl. *Zym*, incl. *Total CellCount*)	Train	96.18 ± 1.42	96.53 ± 1.21	96.18 ± 1.42	96.11 ± 1.45
	Validation	54.85 ± 9.61	60.69 ± 10.71	54.20 ± 8.49	54.49 ± 9.87

a The table shows the accuracy of a single TM trained to discriminate between five classes (No growth, Gram-negative, streptococcal, coagulase-negative *Staphylococcus*, and other Gram-positive bacteria) at once, using the whole biomarkers dataset and a subset of soluble biomarkers (excluding *Zym* and including *TotalCellCount*). TM accuracy was determined for 20 logical clauses per class across 4 biomarker ranges and a total of 39 200 learning automata. The table reports the mean values of training and validation results, as well as their standard deviation calculated over five folds of the stratified *K*-fold cross-validation.

For comparison, ANNs of a comparative complexity with 40 000 hidden nodes achieved an average validation accuracy of only 36.62 ± 8.49% (Supplementary Table S5, available as supplementary data at *Bioinformatics Advances* online). Combined with 100% training accuracy this indicated model overfitting. Whilst providing encouraging proof of concept for the validity of a TM-based approach, the multi-class accuracy fell short of clinical requirements. The limited size of the training set and the complex interdependencies among biomarkers constrained the effectiveness of early-stopping or regularization strategies, inspiring us to explore alternative strategies for improving model generalization.

### 3.2 Hierarchical binary classification and performance of the Tsetlin Machine

To deal with the unsatisfactory accuracy of multi-class classification we next adopted a binary classification approach that focussed on discriminating between a certain class of bacterial infection and other cases of peritonitis, and which had already returned promising results for support vector machines (Zhang *et al.* 2017). We improved it further by structuring it in a hierarchical stepwise manner, resembling a binary decision tree attempting to predict the causative organism in the following order (Fig. 1):

- Discrimination between episodes of peritonitis that yielded no microbiological culture result (‘no growth’; *n* = 19) and episodes where a bacterial pathogen was identified (‘culture-positive’; *n* = 63).
- Within the culture-positive group, discrimination between episodes caused by Gram-negative bacteria (*Acinetobacter baumannii*, *Enterobacter* spp., *Escherichia coli*, *Morganella morganii*, *Proteus vulgaris*, *Pseudomonas aeruginosa* and others; *n* = 17) and episodes caused by Gram-positive bacteria (*n* = 46).
- Within the confirmed infections caused by Gram-positive bacteria, discrimination between episodes caused by streptococcal organisms (*Streptococcus* spp. and *Enterococcus* spp.; *n* = 15) and episodes caused by other, non-streptococcal Gram-positive bacteria (*n* = 31).
- Within the confirmed infections caused by non-streptococcal Gram-positive bacteria, discrimination between episodes caused by coagulase-negative *Staphylococcus* (CNS; *n* = 21) and episodes caused by other Gram-positive bacteria (*Staphylococcus aureus*, *Corynebacterium* spp. and others; *n* = 10).

The hierarchical binary scheme we employed aligns with microbial taxonomy and was directly informed by results of the microbiological analysis of the effluent performed during clinical diagnosis of peritonitis. Each binary decision therefore corresponded both to established pathogen groupings and to known immune‐detection mechanisms studied in peritoneal dialysis patients. Employing a hierarchical binary classification facilitated individual optimization of TM hyperparameters at each classification step, resulting in significant accuracy improvements over a flat multiclass classification approach when using a specialized TM with its learning sensitivity *S* optimized for maximum performance. The remaining hyperparameters (the number of logical clauses per class *C *= 20 and the voting threshold *T *= 3) were kept constant across all TMs.

Three quantitative ranges per biomarker at each classification step were usually sufficient to reliably distinguish between different types of bacterial infection; using only two ranges failed to ensure 99% training and 90% validation accuracy on average, while employing more than four ranges added unnecessary redundancy and data/computational overheads (Table 2). Notably, starting with four semi-quantitative ranges used to Booleanize biomarker values, the TMs persistently achieved a perfect 100% accuracy on both the training and validation sets in at least one fold of the stratified five-fold cross-validation used at each classification step. The best-performing TMs were selected and then used in the rest of the present study to identify and visualize biomarker fingerprints associated with each bacterial infection.

**Table 2.** Train and validation accuracy of TMs at different stages of the hierarchical binary classification depending on the Booleanization precision.^a^

No.	Classification step	Data split	TM accuracy (%)			ANN accuracy (%)
			2 ranges (6400 TAs)	3 ranges (9600 TAs)	4 ranges (12 800 TAs)	
1	No growth versus Culture-positive cases	Train	96.04 ± 2.09	98.40 ± 2.25	99.20 ± 1.17	100
		Validation	87.72 ± 6.66	93.82 ± 6.85	92.57 ± 7.30	81.84 ± 7.46
2	Gram-positive versus Gram-negative bacteria	Train	94.02 ± 1.64	99.72 ± 0.56	100	100
		Validation	89.10 ± 7.80	93.72 ± 3.15	92.05 ± 4.88	69.87 ± 8.86
3	Gram-positive streptococcal versus non-streptococcal bacteria	Train	97.15 ± 2.12	99.20 ± 0.98	100	100
		Validation	88.89 ± 7.03	93.33 ± 5.44	93.56 ± 8.79	71.56 ± 9.26
4	Coag.-neg. *Staphylococcus vs* other Gram-pos. bacteria	Train	98.24 ± 2.35	100	100	92.90 ± 4.75
		Validation	97.14 ± 5.71	97.14 ± 5.71	100	67.62 ± 10.71

a The table shows how the accuracy of a TM with 20 logical clauses per class depends on the number of semi-quantitative intervals used to Booleanize values of soluble biomarkers (excluding *Zym* and including *TotalCellCount*) at each classification step. The rightmost column reports the accuracy achieved by an (ANN) of comparable complexity [13 000 neurons versus Tsetlin automata (TAs)], which was used as a benchmark value. The table reports the mean values of training and validation accuracy, as well as their standard deviation calculated over five folds of the stratified *K*-fold cross-validation used at each classification step.

Of note, TMs sustained high accuracy by tuning its learning sensitivity *S* at each hierarchical step. Optimal *s*-values were identified experimentally for each binary classification step (Table 3). Increasing *S* at lower classification levels sharpened clause specificity, enabling the TMs to capture more fine-grained distinctions in immune fingerprints, and thus maintained robust performance as opposed to ANNs (Supplementary Table S6, available as supplementary data at *Bioinformatics Advances* online). In fact, when using four semi-quantitative Booleanization ranges of biomarker values TMs considerably outperformed ANNs of comparable complexity, particularly in distinguishing Gram-positive from Gram-negative bacteria, and coagulase-negative *Staphylococcus* from other non-streptococcal Gram-positive bacteria. TMs also exhibited smaller standard deviations of training and validation accuracies across the five stratified folds (Table 3, Supplementary Table S6, available as supplementary data at *Bioinformatics Advances* online). This might be explained by the fact that TMs operate by constructing propositional logic clauses over Booleanized (discretized) input features, learning which semi-quantitative ranges each biomarker consistently falls into and how these bin-patterns co-occur for each class. In contrast, ANNs model each biomarker’s exact real-valued contribution via learned weights and nonlinear activations. As a result, TMs are inherently more tolerant of noisy, void or imprecise measurements, as well as imputed or partially missing values, and can therefore outperform ANNs when input data exhibit such imperfections.

**Table 3.** Train and validation performance of TMs at different stages of the hierarchical binary classification using four semi-quantitative Booleanization ranges of biomarker values.^a^

No.	Classification step	(C, T, *s*)	Data split	TM (12 800 TAs) performance (%)			
				Accuracy	Precision	Recall	F1 Score
1	No growth versus Culture-positive cases	(20, 3, 2.4)	Train	99.20 ± 1.17	99.24 ± 1.10	99.20 ± 1.17	99.20 ± 1.17
			Validation	92.57 ± 7.30	88.99 ± 10.17	90.96 ± 9.60	89.75 ± 9.90
2	Gram-positive versus Gram-negative bacteria	(20, 3, 3)	Train	100	100	100	100
			Validation	92.05 ± 4.88	95.18 ± 2.88	85.83 ± 8.16	88.66 ± 6.94
3	Gram-positive streptococcal versus non-streptococcal bacteria	(20, 3, 4)	Train	100	100	100	100
			Validation	93.56 ± 8.79	95.89 ± 3.37	90.00 ± 8.16	91.79 ± 6.70
4	Coag.-neg. *Staphylococcus vs* other Gram-pos. bacteria	(20, 3, 5)	Train	100	100	100	100
			Validation	100	100	100	100

a The table shows the average TM performance (accuracy, precision, recall, and F1 score) over five folds of the stratified *K*-fold cross-validation used at each classification step and reports used TM hyperparameters: *C*, *T* and *s*. In at least one fold, the TMs consistently achieved 100% accuracy on both the training and validation sets. Increasing learning sensitivity *S* at lower classification levels sharpened clause specificity, enabling the TMs to capture more fine-grained distinctions in biomarker patterns (immune fingerprints) and thus maintain robust performance as contrast to ANNs (see Table 2).

### 3.3 Interpretability and visualization of Tsetlin Machine clauses

The major advantage of TMs as compared to other ML algorithms is their natural interpretability and explainability. TMs make predictions based on a set of logical rules (clauses) generated during training, which explain the decision-making and can be verified by specialists. A fragment of such rules created by the TMs to predict the presence of Gram-negative bacteria in patients presenting with acute peritonitis is shown as example in Supplementary Fig. S2, available as supplementary data at *Bioinformatics Advances* online. These rules are represented in the form of conjunctive statements that include specific input features or their negations, thus creating a set of persistent sub-patterns (immune fingerprints) of the target class.

Although these rules were machine readable and easily interpretable, in their raw form they might still be difficult for humans to comprehend and explain, especially if the number of clauses and Boolean features involved was large. To solve this issue, we proposed a clause visualization framework, which represented each clause as a biomarker-wise mask or stencil. For instance, Supplementary Fig. S3, available as supplementary data at *Bioinformatics Advances* online presents an example of interpreting a clause stencil supporting a decision in favour of Gram-negative bacterial infection; the interpretation of TM clauses for other bacterial classes follows the same principle. Each row of this stencil corresponded to a certain biomarker, while each biomarker was represented by a group of bits/pixels corresponding to different value ranges or concentration levels. These ranges were identified for each biomarker during the Booleanization step as part of data pre-processing.

A blue pixel meant that the biomarker value *must* be within that specific range to match the clause rule. Red meant that the biomarker value *must not* be in that range. Finally, white meant ‘ambivalent’, i.e. the biomarker value *may or may not* be in that range. An individual clause could thus be seen as a class template generalizing certain common features of class samples from the training dataset. Each clause formulated a rule by identifying general patterns shared among a subset of patient samples within the same class, for which the clause would output *True* (Supplementary Fig. S3, available as supplementary data at *Bioinformatics Advances* online). Collectively, the team of TM clauses determined the type of bacterial infection by evaluating the number of clauses that supported each classification hypothesis.

### 3.4 Tsetlin Machine inference and decision-making

**Figure 2.**
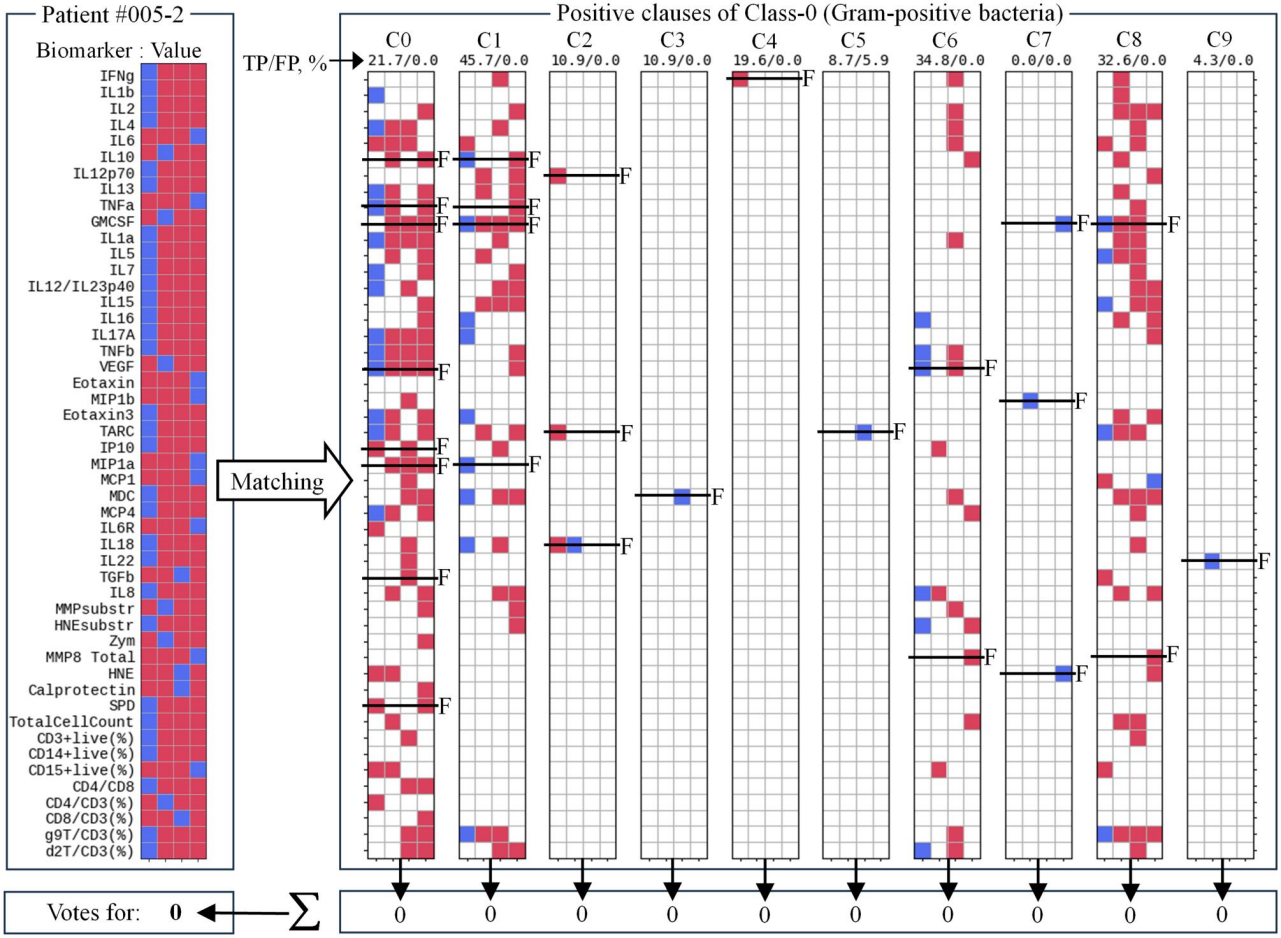
Example of TM inference supporting decision-making in favour of Gram-positive bacterial infection. The figure shows the inference process using TM_2_ clauses trained to recognize Gram-positive (Class-0) bacterial infection by matching the patient sample against clause stencils followed by clause output summation and voting. Here, none of the clauses supported the hypothesis. *F*, biomarkers whose values lay outside the target range specified by the clauses, indicating the corresponding conjunct was *False*. Accuracy labels above each clause show the percentage of True-Positive (TP) and False-Positive (FP) predictions of individual clauses.

**Figure 3.**
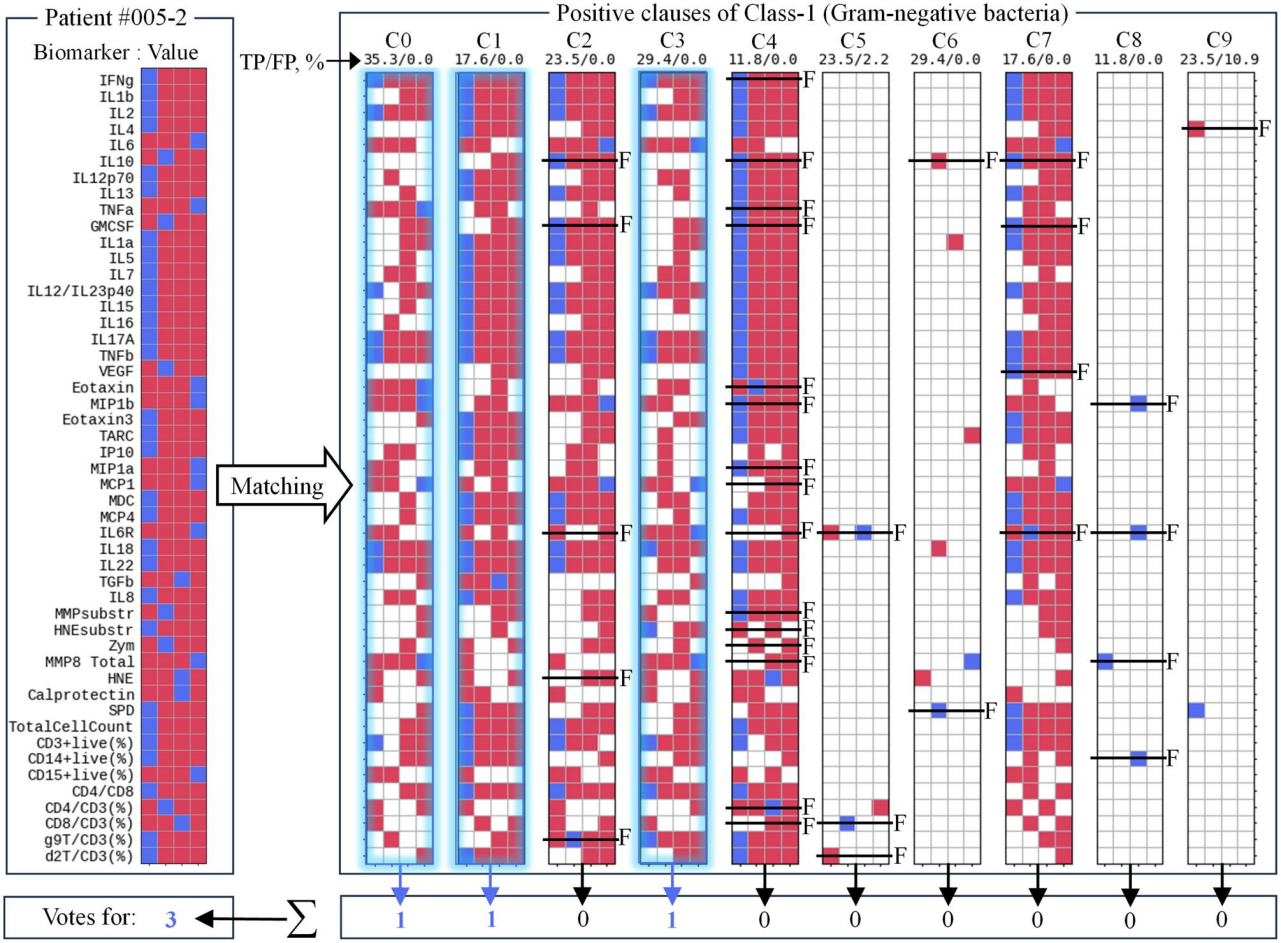
Example of TM inference supporting decision-making in favour of Gram-negative bacteria infection. The figure shows the inference process using TM_2_ clauses trained to recognise Gram-negative (Class-1) bacteria by matching the patient sample against clause stencils followed by clause output summation and voting. Here, 3 out of 10 clauses supported the hypothesis. *F*, biomarkers whose values lay outside the target range specified by the clauses, indicating the corresponding conjunct was *False*. Accuracy labels above each clause show the percentage of True-Positive (TP) and False-Positive (FP) predictions of individual clauses.

During TM inference, the input data sample was matched against all clauses of all classes. If a data sample perfectly aligned with the clause stencil, the clause would output *True*, indicating that the clause cast a vote suggesting the sample belonged to the designated class. Otherwise, the clause would output *False*, which means it abstained from voting. The class with the maximum sum of clause votes was returned as TM prediction. In this sense, TM inference based on clauses voting for and against each class fostered collaborative decision-making and ensured a thorough and holistic assessment. As example, Figs 2 and 3 illustrate the inference process that enabled differentiation between Gram-negative and Gram-positive bacterial infections.

Figure 2 shows how the data sample from patient 005–2 was matched against 10 positive TM clauses trained to recognize and vote for Class-0 (Gram-positive bacteria). In this particular example, there was no match with any of these 10 positive clauses. Biomarkers whose values lay outside the target range specified by the clauses were labelled ‘*F*’, indicating that the corresponding conjunct was *False*. Consequently, all 10 clauses abstained from supporting the decision that patient 005–2 was infected with Gram-positive bacteria. The results of matching the same patient sample against positive clauses of Class-1 (Gram-negative bacteria) are shown in Fig. 3. The data sample matched three clauses (C0, C1, and C3) out of 10. Ultimately, with three votes to zero, the patient 005–2 was classified as having a Gram-negative bacterial infection. Voting margin could be seen as an additional measure of confidence offered by TMs in decision-making. TMs thus offered a useful mechanism of logical clauses which justified decision-making and could be easily interpreted, visualized and verified.

### 3.5 Identification of key biomarkers and clause minimization

TMs facilitate the identification and ranking of key features (in this case, biomarkers) based on their impact on decision-making, whilst preserving logical relations between them. The initial step involves calculating the frequency with which each feature appears in the positive clauses of each class and negative clauses of the opposing class(es). Features that occur equally often in the clauses of either class carry the least discriminatory power; the most significant are those that are unique to each class.

Here, the importance of each feature (a feature rank) was calculated by taking the absolute difference between the sum of occurrences of the feature in logical clauses that predicted the target class, and the sum of appearance of the feature in clauses in clauses that predicted the opposing class. A higher feature rank indicated that the feature had a strong association with one class over the other, making it highly influential in distinguishing between the two classes. Since each feature *x_i_* encoded a certain semi-quantitative interval for a particular biomarker, once the key features were identified, they were mapped back to their corresponding biomarkers. Finally, we assembled the minimized biomarker set by starting with the single most influential biomarker and progressively including other top-ranked biomarkers until the TM accuracy reached the specified threshold.

Supplementary Figure S4, available as supplementary data at *Bioinformatics Advances* online presents the minimal subsets of biomarkers that achieved an overall prediction accuracy of 90%, 95%, and 100%, respectively, using the top-performing TM from five-fold cross-validation at each classification step when each biomarker value was encoded into four semi-quantitative intervals. Perhaps not surprisingly, achieving higher accuracy required more biomarkers to be taken into consideration. For example, distinguishing between culture-positive infections and cases of no microbiological growth could be done with 90% accuracy using only 11 biomarkers. Achieving 95% required 13 biomarkers, while considering 21 biomarkers allowed to reach 100% accuracy.

At the same time, the later stages of hierarchical binary classifications required progressively fewer biomarkers. Taken together, these findings demonstrated that each type of microbiologically confirmed infection was associated with a distinct set of biomarkers that set it apart from infections with other organisms. For instance, accurate prediction of culture-positive episodes of peritonitis required cellular parameters such as the total cell count (*TotalCellCount*), the proportion of neutrophils amongst infiltrating immune cells [*CD15+live(%)*] and the proportion of Vδ2^+^ T cells amongst T cells (*d2T/CD3*), as well as a distinct set of cytokines and chemokines (Supplementary Fig. S4, available as supplementary data at *Bioinformatics Advances* online).

### 3.6 Focus on soluble biomarkers for better clinical applicability

**Figure 4.**
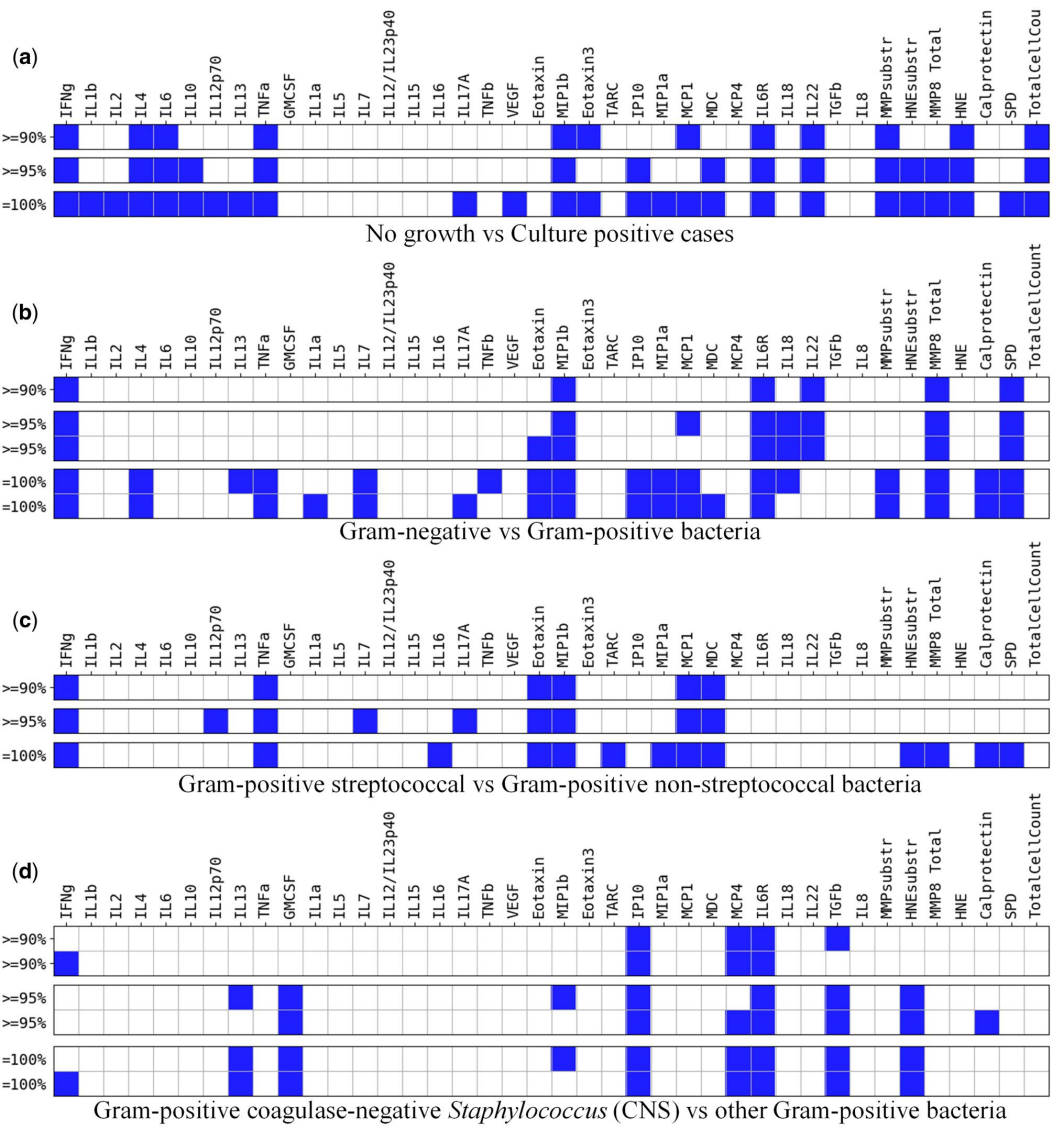
Minimized sets of soluble biomarkers (excluding *Zym* and including *TotalCellCount*). Figure shows the minimized set of soluble biomarkers needed to make predictions at different classification stages with the target accuracies of 90%, 95%, and 100% for the case where each biomarker value is represented by four semi-quantitative ranges.

To enhance the clinical applicability of our research, we next attempted to streamline the dataset by reducing the number of semi-quantitative ranges used to quantize biomarker values, and by considering only soluble immune mediators, i.e. biomarkers that can easily be quantified using ELISA-based techniques. We therefore excluded the measurement of matrix metalloproteinase (MMP)-9 activity using gelatin zymography (*Zym*) as well as all flow cytometric characterizations of immune cell subsets, methods which would be too complex for routine diagnostic application (Supplementary Table S3, available as supplementary data at *Bioinformatics Advances* online). As only cellular biomarker, we did keep the total cell count in the ‘soluble’ dataset (*TotalCellCount*) as this parameter is determined routinely in the clinic and thus readily accessible to guide treatment decisions. Importantly, using this reduced set of biomarkers, correct classification was still possible (Fig. 4).

However, distinguishing between culture-positive infections and cases of no growth with 90%, 95%, and 100% accuracy now required more individual biomarkers than before—12, 15, and 25, respectively. Overall, our calculations demonstrated that reducing the number of semi-quantitative ranges lowered the dimensionality of the input space, enhanced clause interpretability and might simplify future biomarker tests. However, this simultaneously increased the number of biomarkers required to achieve the desired target accuracy. As such, these findings underscored the importance of cellular biomarkers that contributed key information needed for accurate classification using the smallest possible biomarker combination. Table 4 summarizes the minimal number of biomarkers for both the full original dataset versus the reduced but clinically more tractable dataset consisting of only soluble biomarkers, in relation to the numbers of semi-quantitative intervals used to Booleanize and discretize continuous biomarker values (Supplementary Figs S4–S6, available as supplementary data at *Bioinformatics Advances* online).

**Table 4.** Minimal number of biomarkers required to achieve the target accuracy for different datasets and numbers of semi-quantitative value ranges.^a^

No.	Classification step	Overall classification accuracy	Full dataset (40 soluble + 9 cellular biomarkers)	Soluble biomarkers (excl. *Zym*, incl. *TotalCellCount*)		
			Four ranges	4 ranges	3 ranges	2 ranges
1	No growth *vs* Culture-positive cases	≥90%	11 (9 soluble + 3 cellular)	12	12	19
		≥95%	13 (10 soluble + 3 cellular)	15	16	24
		≥99%	21 (16 soluble + 5 cellular)	25	33	33 (96.3% max. accuracy)
2	Gram-positive *vs* Gram-negative bacteria	≥90%	6 (6 soluble)	6	12	17
		≥95%	8 (6 soluble + 2 cellular)	8	16	20
		≥99%	16 (11 soluble + 5 cellular)	17	22	20 (96.8% max. accuracy)
3	Gram-positive streptococcal *vs* non-streptococcal bacteria	≥90%	5 (3 soluble + 2 cellular)	6	10	11
		≥95%	9 (7 soluble + 2 cellular)	9	13	13
		≥99%	12 (9 soluble + 3 cellular)	13	18	15 (97.8% max. accuracy)
4	Coag.-neg. *Staphylococcus vs* other Gram-positive bacteria	≥90%	3 (3 soluble incl. *Zym*)	5	6	8
		≥95%	6 (4 soluble + 2 cellular)	7	7	16
		≥99%	7 (4 soluble + 3 cellular)	8	8	21

a The table reveals a trade-off between the biomarker values granularity (i.e. the number of semi-quantitative ranges used to quantise biomarkers) and the number of biomarkers needed to achieve the target accuracy. The exact identity of the corresponding biomarkers is shown in Fig. 4 and in Supplementary Figs S4–S6, available as supplementary data at *Bioinformatics Advances* online.

### 3.7 Clause pruning

In addition to minimization of the number of biomarkers used in a logical clause, the set of clauses can also be minimized, or pruned (Liu *et al.* 2021), based on clause precision and their contribution to the accurate inference. Precision was estimated by the ratio between *True* (TP) and *False* (FP) predictions made by each individual clause (i.e. a logical inference rule): TP/(TP+FP), after each training round and after testing. The proportion between TP and FP is shown at the top of each TM clause in Figs 2 and 3. Clauses with the minimal contribution to the true predictions (*e.g.* clauses C7 and C9 in Fig. 2) or with a considerable portion of false predictions (*e.g.* clause C5 in Fig. 2 and clause C9 in Fig. 3) could be pruned from the TMs. Clause pruning began with the least precise clauses and continued iteratively until TM performance stayed above the specified accuracy threshold (i.e. 90%, 95% or 99%).

**Figure 5.**
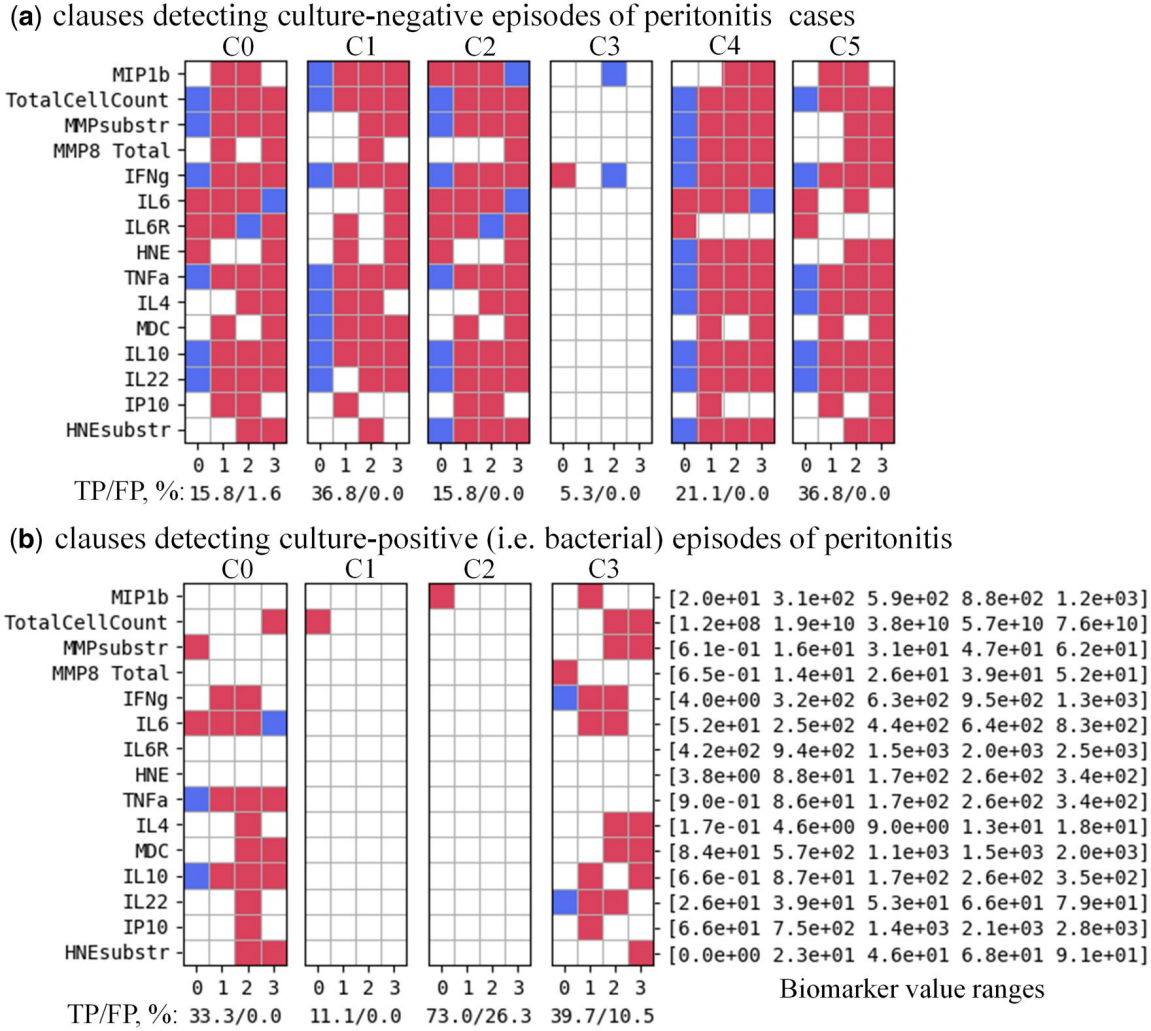
Minimized set of TM clauses and soluble biomarkers used to discriminate between episodes of peritonitis that yielded no microbiological growth (a) versus culture-positive episodes (b) with 95.12% accuracy for the case where each biomarker value was represented by four semi-quantitative ranges. Accuracy labels under each clause show the percentage of True-Positive (TP) and False-Positive (FP) predictions of individual clauses. A balance between clause generalization and specialization, which defines a ratio between *True* and *False* predictions made by each clause is controlled by TM hyper-parameters (Tarasyuk *et al.* 2023) and affects the overall classification accuracy.

**Figure 6.**
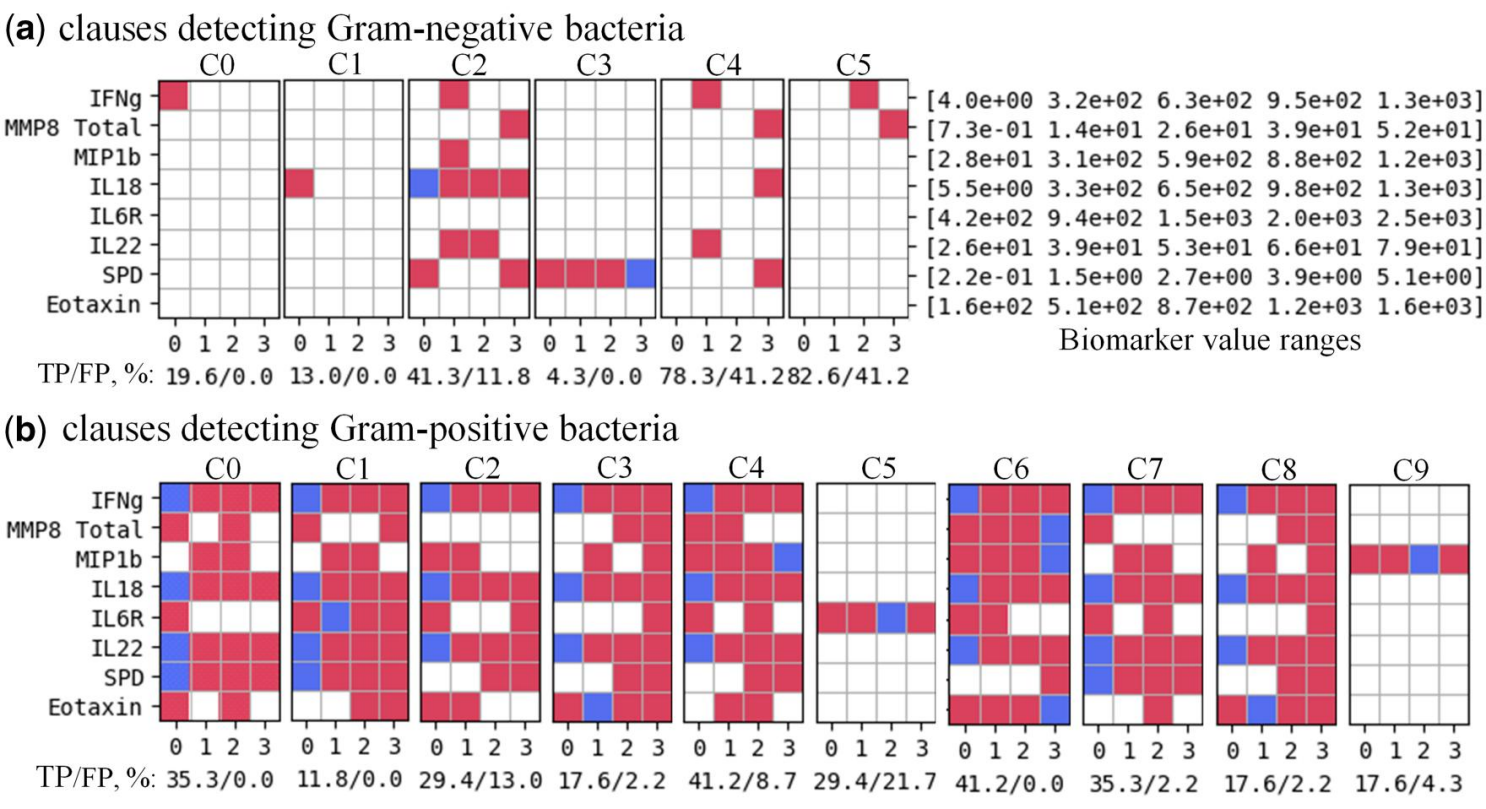
Minimized set of TM clauses and soluble biomarkers used to discriminate between episodes caused by Gram-negative (a) versus Gram-positive bacteria (b) within the culture-positive group of patients with 95.24% accuracy for the case where each biomarker value was represented by four semi-quantitative ranges. Accuracy labels under each clause define the percentage of True Positive (TP) and False-Positive (FP) predictions of individual clauses.

**Figure 7.**
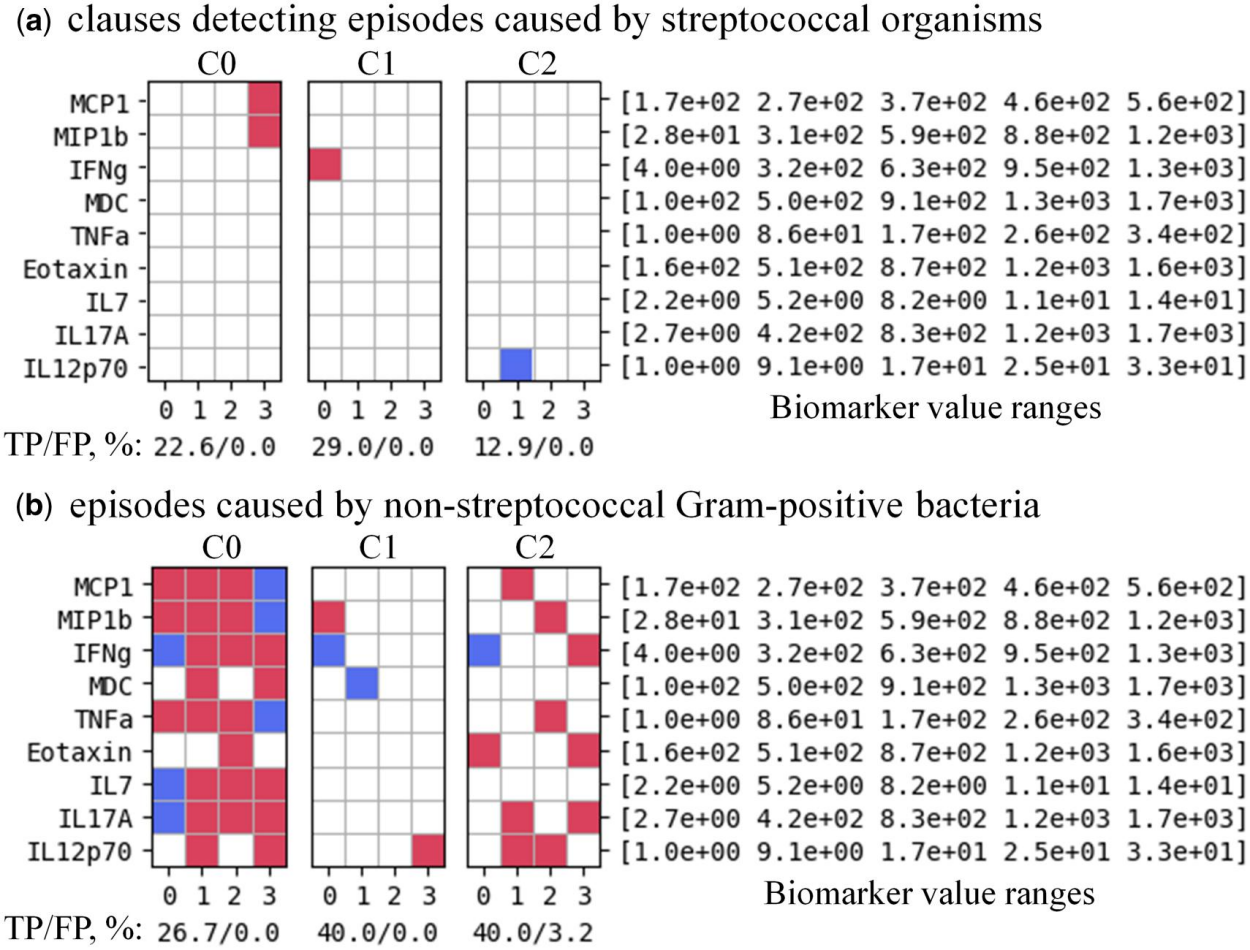
Minimized set of TM clauses and soluble biomarkers used to discriminate between episodes caused by streptococcal organisms (a) versus episodes caused by non-streptococcal Gram-positive bacteria (b) with 95.65% accuracy for the case where each biomarker value was represented by four semi-quantitative ranges. Accuracy labels under each clause define the percentage of True-Positive (TP) and False-Positive (FP) predictions of individual clauses.

**Figure 8.**
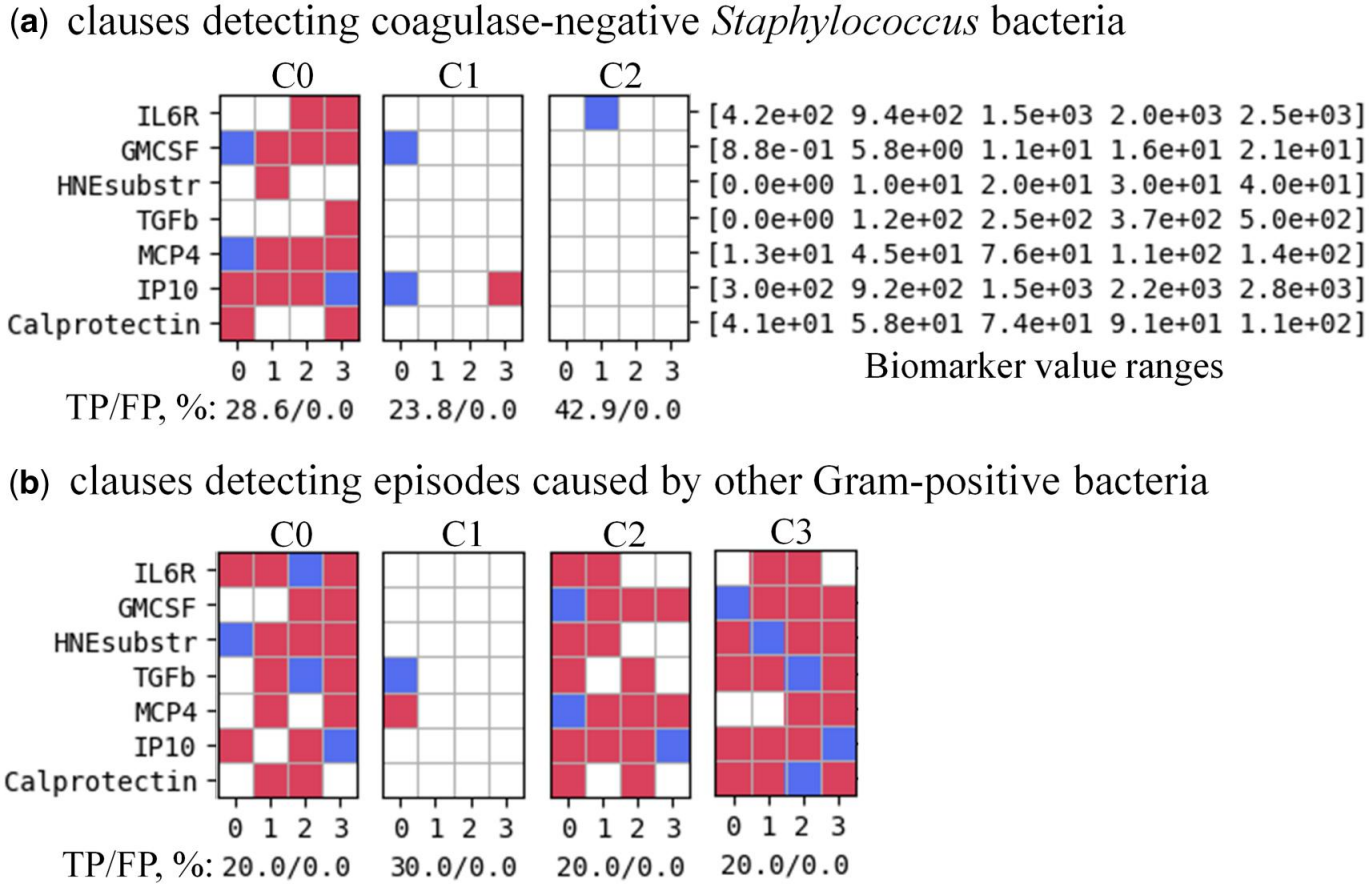
Minimized set of TM clauses and soluble biomarkers used to discriminate between episodes caused by coagulase-negative *Staphylococcus* versus episodes caused by other Gram-positive bacteria with 96.77% accuracy for the case where each biomarker value was represented by four semi-quantitative ranges. Accuracy labels under each clause define the percentage of True-Positive (TP) and False-Positive (FP) predictions of individual clauses.

Figures 5–8 present the optimized sets of TM clauses used at each classification step after minimizing the number of biomarkers and removing less efficient clauses.

For example, the number of soluble biomarkers was reduced from 40 to 8, followed by further pruning of the clauses from 20 per class to just 6 clauses for detecting Gram-positive bacteria and 10 clauses for identifying Gram-negative bacteria (Fig. 6).

**Figure 9.**
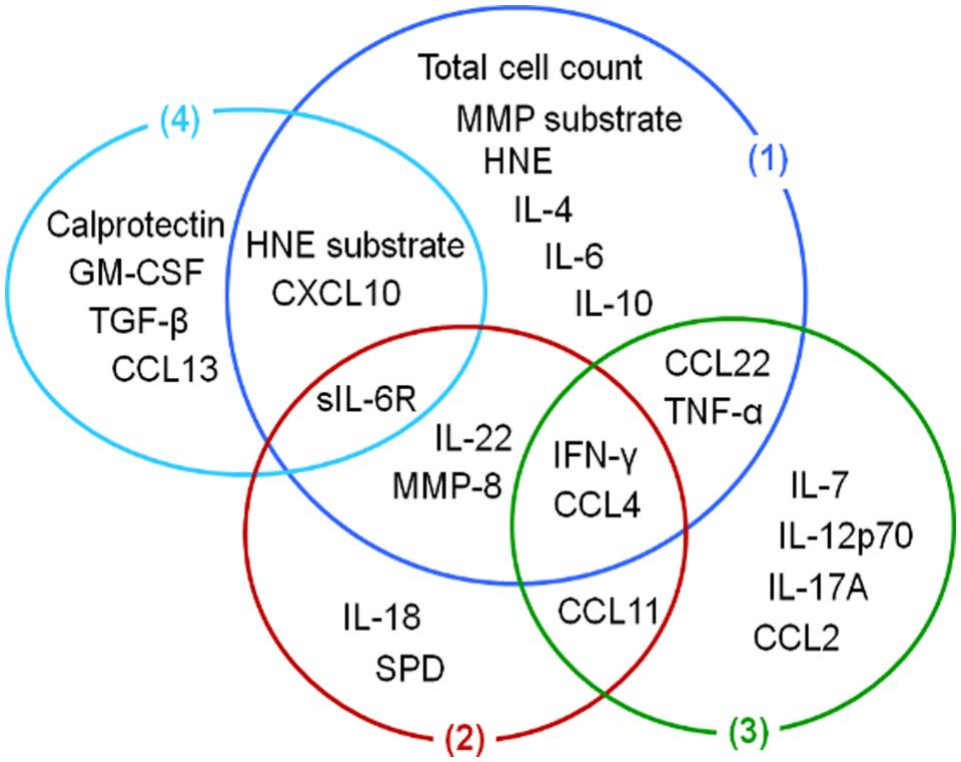
Minimized set of biomarkers defining immune fingerprints associated with peritonitis caused by different types of bacteria with an accuracy ≥95%. (1) discrimination between episodes of peritonitis that yielded no microbiological growth versus culture-positive episodes; (2) discrimination between episodes caused by Gram-negative bacteria within the culture-positive group of patients; (3) discrimination between episodes caused by streptococcal organisms versus episodes caused by non-streptococcal Gram-positive bacteria; (4) discrimination between episodes caused by coagulase-negative *Staphylococcus* versus episodes caused by other Gram-positive bacteria.

Despite considerable minimization, the pruned model could still successfully distinguish between Gram-positive and Gram-negative bacteria with 95% accuracy. Similar TM optimization could be applied to all other steps of the hierarchical classification approach used in this study (Figs 5–8), yielding a comprehensive and overlapping set of biomarkers that defined immunologically distinct local responses during early peritonitis in patients presenting with acute symptoms (Fig. 9). In our earlier study (Zhang *et al.* 2017), the *caret* package (v. 2.27) in R, which implements the Recursive Feature Elimination (RFE) method, was applied to identify the most influential biomarkers for SVM, RF and ANN models. The resulting feature subsets shown in Fig. 9 overlapped largely (by 84.6%) with the top biomarkers associated with different types of causative organisms reported previously (Zhang *et al.* 2017), further validating the present TM findings.

**Figure 10.**
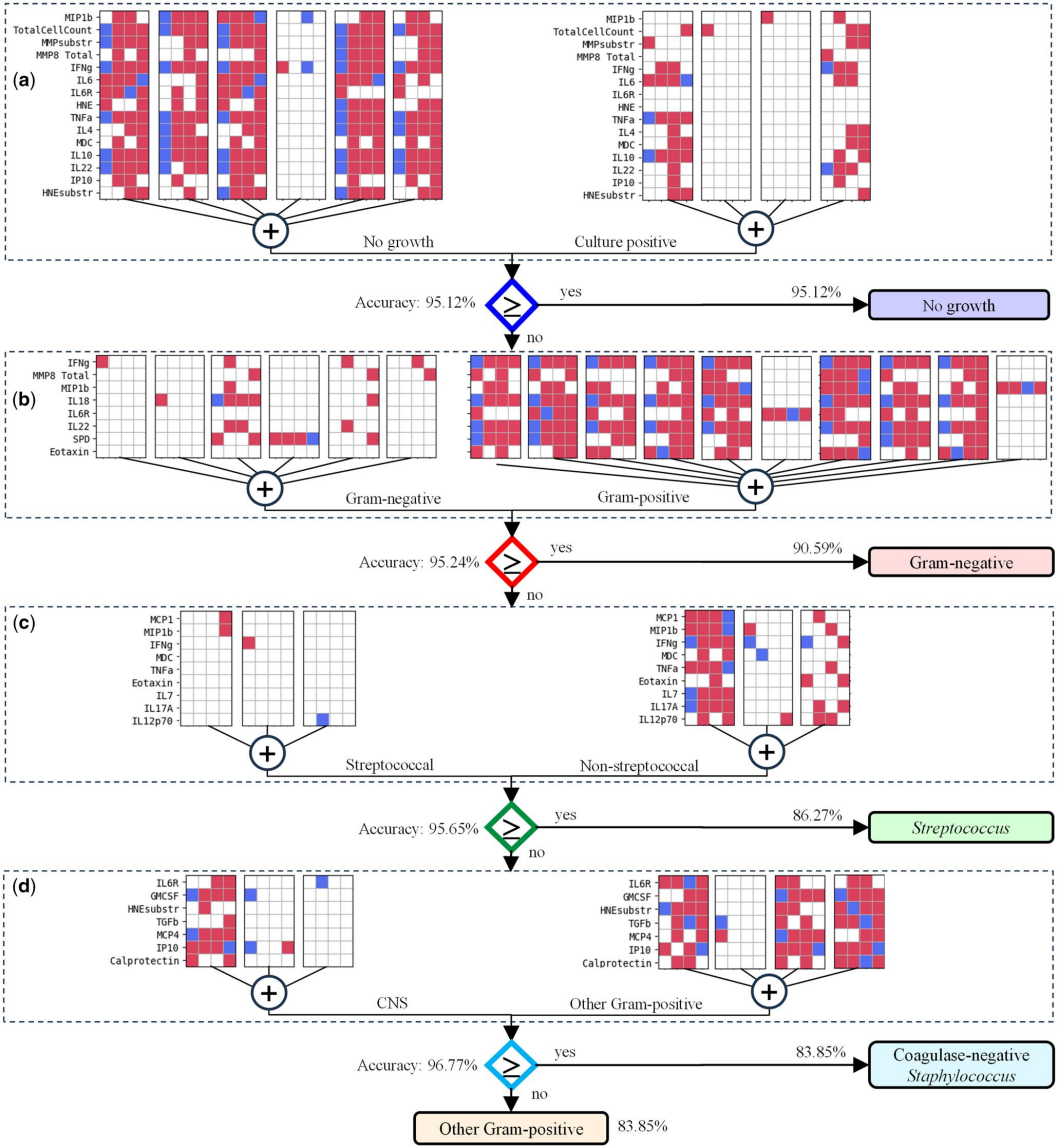
Decision-making tree implementing the hierarchical binary classification of acute peritonitis using the minimized set of soluble biomarkers and TM logical clauses ensuring 95% accuracy at each classification step: (a) no growth versus culture-positive cases; (b) Gram-negative vs Gram-positive bacteria; (c) Gram-positive streptococcal versus Gram-positive non-streptococcal bacteria; and (d) Gram-positive coagulase-negative *Staphylococcus* (CNS) versus other Gram-positive bacteria.

The overall decision-making process implementing the hierarchical binary classification of acute peritonitis and using the minimized set of soluble biomarkers and TM logical clauses at each classification step is shown in Fig. 10. Accuracy after each step of the hierarchical classification would be no worse than the product of the prediction accuracies achieved until that point. Thus, accuracy of predicting a specific group of bacterial infections could be controlled by increasing or decreasing the number of biomarkers to be determined at a certain layer, as deemed necessary or appropriate (Fig. 4).

## 4 Discussion

In this study, we demonstrate the potential of TMs as an effective ML model for analysing local immune responses in patients with life-threatening bacterial infection. By leveraging the logic-based framework of TMs, we successfully identified pathogen-specific immune fingerprints, represented as logical clauses, which are both easily interpretable and actionable for clinical decision-making. These logical rules provide insights into the distinctive biomarker profiles associated with different types of bacterial infections, enabling rapid and precise classification even before conventional microbiological results are available.

At the core, our study reaffirms the notion that different pathogens elicit qualitatively and quantitatively distinct immune responses, even when infecting the same anatomical location and causing indistinguishable clinical symptoms (Lin *et al.* 2013, Zhang *et al.* 2017). This might not come as a surprise considering that each bacterium expresses a unique set of pathogen-associated molecular patterns, antigens and virulence factors interacting with a myriad of pattern recognition factors and antigen receptors of the immune system (Kroemer *et al.* 2024, Medzhitov and Iwasaki 2024). For instance, the outer membrane of Gram-negative bacteria contains lipopolysaccharides, highly immunogenic molecules that trigger inflammatory responses via Toll-like receptor 4 (TLR4) expressed on monocytes, macrophages, dendritic cell, other immune cells and many tissues. Gram-positive bacteria are free of lipopolysaccharides but can be sensed via TLR2 (Colmont *et al.* 2011), thus defining a clear mechanism how the body discriminates between the two main groups of bacteria. Similarly, TLR5 recognizes flagellin, a principal component of flagella carried by bacteria such as *Salmonella* spp., *Pseudomonas aeruginosa*, *Listeria monocytogenes* and some strains of *E. coli* but not others (Liuzzi *et al.* 2015). Many microbial organisms also express the highly potent metabolite (*E*)-4-hydroxy-3-methyl-but-2-enyl pyrophosphate (HMB-PP), which specifically activates a small subset of T lymphocytes expressing a Vγ9/Vδ2 T cell receptor; notable HMB-PP deficient pathogens of clinical relevance in PD include streptococcal and staphylococcal bacteria (Liuzzi *et al.* 2015). Together, the unique combination of such immunogenic molecules defines each microorganism and is likely to result in immunologically distinct activation pathways.

We believe that the presence of pathogen-specific immune responses has never been documented in infected patients as clearly as in the current study, demonstrating distinct local immune responses in patients with severe infection. Of note, some of these features were particularly relevant for predicting the presence of microbiologically confirmed bacterial organisms versus cases of no growth, including the total cell count, MMP activity (*MMPsubstr*) and levels of human neutrophil elastase (HNE), IL-4, IL-6, and IL-10 in the peritoneal effluent. Others were found to play a role in predicting specific types of bacteria, such as levels of IL-18 and surfactant protein D for the discrimination between Gram-negative and Gram-positive infections; levels of IL-7, IL-12p70, IL-17A, and CCL2 for the discrimination between streptococcal and non-streptococcal Gram-positive infection; and levels of calprotectin, GM-CSF, TGF-β, and CCL13 for the prediction of coagulase-negative *Staphylococcus* infections versus other types of Gram-positive infections. Several biomarkers featured in immune fingerprints associated with more than one type of peritonitis, suggesting a particularly useful role for the differential diagnosis of PD patients, namely HNE activity (*HNEsubstr*) and effluent levels of IFN-γ, TNF-α, IL-22, sIL-6R, MMP-8, CCL4, CCL11, CCL22, and CXCL10. Reassuringly, despite using entirely different statistical methodologies, these patterns were remarkably similar to our earlier analyses associating TNF-α with culture-positive episodes and IL-22 and CXCL10 with Gram-positive infections (Lin *et al.* 2013), as well as the importance of the total cell count for culture-positive episodes, IFN-γ and IL-17A for non-streptococcal Gram-positive infections, and sIL-6R for staphylococcal infections (Zhang *et al.* 2017). Differences with regard to other biomarkers may in part be due to the fact that TMs work with Booleanized semi-quantitative input values rather than precise measurements as used in previous studies, and that we here used a stepwise classification of patients.

Our findings highlight the capability of TMs to address critical challenges in biomedical ML, such as interpretability and efficiency. Unlike traditional ‘black boxes’ models, TMs offer a transparent decision-making process, which is essential in clinical settings where understanding and trust in predictive models are paramount, while maintaining accuracy that is competitive, and under certain conditions, superior, to other ML classifiers such as SVM, RF and ANN as utilized in our previous work (Zhang *et al.* 2017). Additionally, the ability of TMs to operate on Booleanized, semi-quantitative data underscores their suitability for mass clinical use, particularly in combination with rapid, accessible testing methods like lateral flow tests. Recent advances in lateral flow technology have already demonstrated the ability to determine multiple levels of analyte concentration (Hu *et al.* 2017, You *et al.* 2018), rather than simply detect the presence (or absence) of an individual biomarker.

In this respect, a recently developed lateral flow test for diagnosis of peritonitis may not only be useful for the detection of early infection based on elevated levels of IL-6 and MMP-8 in PD effluent (Goodlad *et al.* 2020) but in a more quantitative way even contribute to the distinction between culture-positive episodes and cases of no growth, as suggested in the present study.

The hierarchical classification methodology employed in this study achieved high accuracy with minimal biomarker input, emphasizing the strength of TMs in feature reduction and efficient data utilization. This approach not only enhances diagnostic precision but also minimizes the overall number of tests required, reducing both costs and time-to-diagnosis. In conclusion, TMs present a robust framework for decoding and visualizing complex immune responses, offering a promising avenue for real-time, interpretable diagnostics in infectious disease management. Future work should focus on validating this approach in independent cohorts and expanding the application of TMs to different demographics and diverse infectious agents, potentially broadening its utility in clinical diagnostics and personalized medicine.

To accelerate progress, we encourage the broader community to share relevant datasets, whether from peritoneal dialysis or other infection conditions, and to benchmark classifiers based on the Tsetlin Machine against established ‘black box’ methods with post-hoc explanation tools.

On the implementation side, engineering low-cost, multiplexed LFTs producing results ready for logic-based interpretation and further calibrating thresholds would bridge the gap between proof-of-concept and point-of-care use. By sharing our code, rules and biomarker sets, we aim to catalyse collaborative efforts that validate and refine interpretable logic-based classifiers in real-world clinical contexts, ultimately paving the way for rapid, trustworthy diagnostics across a range of infectious conditions.

## Supplementary Material

vbaf140_Supplementary_Data

## Data Availability

All underlying tools and the anonymized data underpinning this publication are available at https://github.com/anatoliy-gorbenko/biomarkers-visualization.
