# Supplementary material for: Prediction of the infecting organism in peritoneal dialysis patients with acute peritonitis using interpretable Tsetlin Machines
Source: Bioinform Adv. 2025 Jun 19;5(1):vbaf140. doi: 10.1093/bioadv/vbaf140 (PMC12237506; doi:10.1093/bioadv/vbaf140)
Supplement: vbaf140_Supplementary_Data [file vbaf140_supplementary_data.pdf]

Prediction of the infecting organism in peritoneal dialysis patients with acute peritonitis using interpretable Tsetlin Machines

Olga Tarasyuk[1], Anatoliy Gorbenko[2], Matthias Eberl[3,4*], Nicholas Topley,[3,4]
Jingjing Zhang[1,3,4], Rishad Shafik[1], Alex Yakovlev[1]

[1]*School of Engineering, Newcastle University,* Newcastle upon Tyne, UK
[2]*School of Built Environment, Engineering and Computing, Leeds Beckett University,* Leeds, UK
[3]*Division of Infection & Immunity, School of Medicine, Cardiff University,* Cardiff, UK
[4]*Systems Immunity Research Institute, Cardiff University,* Cardiff, UK

Supplemental Information

Supplemental Table S1. Basic demographics and microbiological culture results of all patient samples analysed in the present study.

Patient	Gender	Age	Microbiological culture result
No growth			
001-1	M	82.2	No growth
003-1	M	61.2	No growth
003-2	M	61.3	No growth
003-3	M	61.9	No growth
003-5	M	62.1	No growth
011-1	M	63.0	No growth
011-2	M	63.1	No growth
024-2	F	69.7	No growth
026-1	M	74.8	No growth
030-1	F	61.3	No growth
036-2	M	44.4	No growth
038-1	F	31.6	No growth
051-11	F	86.1	No growth
069-1	F	62.4	No growth
163-1	F	64.8	No growth
174-2	F	72.5	No growth
177-1	M	63.9	No growth
181-2	M	74.6	No growth
189-2	F	50.4	No growth
Gram-negative bacteria			
016-1	M	76.3	*Acinetobacter* sp.
189-1	F	50.3	*Acinetobacter baumannii*
175-1	M	79.7	*Enterobacter* sp.
015-1	M	56.1	*Escherichia coli*
020-1	F	50.8	*Escherichia coli*
031-1	F	64.2	*Escherichia coli*
117-6	F	35.7	*Escherichia coli*
132-9	M	77.0	*Escherichia coli*
156-3	M	73.6	*Escherichia coli*
164-2	F	73.3	*Escherichia coli*
196-1	M	67.4	*Enterobacter* sp.
054-3	F	82.3	*Morganella morganii*
005-2	F	68.5	*Proteus vulgaris*
022-1	M	73.2	*Pseudomonas aeruginosa*
029-1	M	75.0	*Pseudomonas aeruginosa*
174-1	F	72.3	*Pseudomonas aeruginosa*, anaerobic Gram-negative bacilli
084-1	M	90.2	Gram-negative bacilli
Gram-positive bacteria, streptococcal organisms			
156-1	M	73.2	Alpha-haemolytic *Streptococcus*
019-1	M	62.5	Alpha-haemolytic *Streptococcus*
039-1	M	83.8	Alpha-haemolytic *Streptococcus*

070-10	M	80.1	Alpha-haemolytic *Streptococcus*
116-6	M	67.7	Alpha-haemolytic *Streptococcus*
004-1	M	70.2	Alpha-haemolytic *Streptococcus*
023-3	M	86.5	Alpha haemolytic *Streptococcus*
013-1	M	75.5	Alpha-haemolytic *Streptococcus*, other *Streptococcus* sp.
040-2	F	61.9	*Enterococcus faecalis*
159-1	M	43.8	*Enterococcus faecium*, group A *Streptococcus*
018-2	M	59.6	Microaerophilic *Streptococcus*
023-1	M	84.1	Microaerophilic *Streptococcus*
227-1	F	58.9	*Streptococcus* B
135-4	F	67.6	*Streptococcus sanguinis*
150-2	M	83.1	Vancomycin-resistant *Enterococcus* (VRE)
Gram-positive bacteria, CNS			
002-1	M	64.4	Coagulase-negative *Staphylococcus*
012-2	M	65.0	Coagulase-negative *Staphylococcus*
025-1	M	70.3	Coagulase-negative *Staphylococcus*
035-1	F	35.5	Coagulase-negative *Staphylococcus*
045-1	F	59.2	Coagulase-negative *Staphylococcus*
068-2B	M	69.9	Coagulase-negative *Staphylococcus*
077-1	F	72.0	Coagulase-negative *Staphylococcus*
080-2	M	50.8	Coagulase-negative *Staphylococcus*
082-4	F	50.4	Coagulase-negative *Staphylococcus*
107-1A	F	61.8	Coagulase-negative *Staphylococcus*
115-3	M	91.4	Coagulase-negative *Staphylococcus*
126-2	M	33.5	Coagulase-negative *Staphylococcus*
150-1	M	83.0	Coagulase-negative *Staphylococcus*
152-4	M	78.0	Coagulase-negative *Staphylococcus*
154-1	F	65.6	Coagulase-negative *Staphylococcus*
167-1	M	44.2	Coagulase-negative *Staphylococcus*
181-1	M	74.3	Coagulase-negative *Staphylococcus*
184-1	M	79.4	Coagulase-negative *Staphylococcus*
184-2	M	79.5	Coagulase-negative *Staphylococcus*
184-4	M	79.7	Coagulase-negative *Staphylococcus*
197-1	F	60.2	Coagulase-negative *Staphylococcus*
Gram-positive bacteria, other			
027-1	F	69.3	*Corynebacterium amycolatum*
037-1	F	47.3	Coryneform bacteria
021-1	M	65.9	Coryneform bacteria, Gram-positive bacilli
012-1	M	62.1	Coryneform bacteria, coagulase-negative *Staphylococcus*
056-1	F	39.1	*Staphylococcus aureus*
039-2	M	84.0	*Staphylococcus aureus*
068-8	M	72.5	*Staphylococcus aureus*
070-1	M	79.0	*Staphylococcus aureus*
098-12	M	50.0	*Staphylococcus aureus*
056-3	F	39.2	*Staphylococcus aureus* (MRSA)

Supplemental Table S2. Demographics of patient samples analysed in the present study. CNS, coagulase-negative *Staphylococcus*; SEM, standard error of the mean.

	No growth		Gram-negative		*Streptococcus* spp.		CNS		Other Gram-positive	
	n	%	*n*	%	*n*	%	*n*	%	*n*	%
Age (mean ± SEM)	63.8 ± 2.9		68.6 ± 3.3		70.6 ± 3.1		65.2 ± 3.4		60.9 ± 4.6	
[18-40]	1	5.3	1	5.9	0	0	2	9.5	2	18.2
[40-50]	1	5.3	0	0	1	6.7	1	4.8	1	9.1
[50-60]	1	5.3	3	17.6	2	13.3	3	14.3	1	9.1
[60-70]	11	57.9	3	17.6	4	26.7	6	28.6	4	36.4
[70-80]	3	15.8	8	47.1	3	20.0	7	33.3	2	18.2
≥80	2	10.5	2	11.8	5	33.3	2	9.5	1	9.1
Sex										
Male	11	57.9	9	52.9	12	80	14	66.7	6	54.5
Female	8	42.1	8	47.1	3	20	7	33.3	5	45.5

Supplemental Table S3. Soluble and cellular immune biomarkers in peritoneal effluent. Methodological details were described before (Zhang et al., 2017). BD, BD Biosciences; MSD, Meso Scale Discovery.

Biomarker	Symbol	Method, manufacturer
IL-1α (pg/ml)	`IL1a`	V-PLEX Cytokine 30-Plex Kit, MSD
IL-1β (pg/ml)	`IL1b`	V-PLEX Cytokine 30-Plex Kit, MSD
IL-2 (pg/ml)	`IL2`	V-PLEX Cytokine 30-Plex Kit, MSD
IL-4 (pg/ml)	`IL4`	V-PLEX Cytokine 30-Plex Kit, MSD
IL-5 (pg/ml)	`IL5`	V-PLEX Cytokine 30-Plex Kit, MSD
IL-6 (pg/ml)	`IL6`	V-PLEX Cytokine 30-Plex Kit, MSD
IL-7 (pg/ml)	`IL7`	V-PLEX Cytokine 30-Plex Kit, MSD
IL-10 (pg/ml)	`IL10`	V-PLEX Cytokine 30-Plex Kit, MSD
IL-12p40 (pg/ml)	`ILp40`	V-PLEX Cytokine 30-Plex Kit, MSD
IL-12p70 (pg/ml)	`ILp70`	V-PLEX Cytokine 30-Plex Kit, MSD
IL-13 (pg/ml)	`IL13`	V-PLEX Cytokine 30-Plex Kit, MSD
IL-15 (pg/ml)	`IL15`	V-PLEX Cytokine 30-Plex Kit, MSD
IL-16 (pg/ml)	`IL16`	V-PLEX Cytokine 30-Plex Kit, MSD
IL-17A (pg/ml)	`IL17A`	V-PLEX Cytokine 30-Plex Kit, MSD
IL-18 (pg/ml)	`IL18`	Single-plex assay, MSD
IL-22 (pg/ml)	`IL22`	Single-plex assay (customised), MSD
sIL-6R (pg/ml)	`IL6R`	Single-plex assay, MSD
IFN-γ (pg/ml)	`IFNg`	V-PLEX Cytokine 30-Plex Kit, MSD
TNF-α (pg/ml)	`TNFa`	V-PLEX Cytokine 30-Plex Kit, MSD
TNF-β (pg/ml)	`TNFb`	V-PLEX Cytokine 30-Plex Kit, MSD
GM-CSF (pg/ml)	`GMCSF`	V-PLEX Cytokine 30-Plex Kit, MSD
TGF-β (pg/ml)	`TGFb`	ELISA, R&D Systems
VEGF (pg/ml)	`VEGF`	V-PLEX Cytokine 30-Plex Kit, MSD
CCL2 (pg/ml)	`MCP1`	ELISA, BD Biosciences
CCL3 (pg/ml)	`MIP1a`	V-PLEX Cytokine 30-Plex Kit, MSD
CCL4 (pg/ml)	`MIP1b`	V-PLEX Cytokine 30-Plex Kit, MSD
CCL11 (pg/ml)	`Eotaxin`	V-PLEX Cytokine 30-Plex Kit, MSD
CCL13 (pg/ml)	`MCP4`	V-PLEX Cytokine 30-Plex Kit, MSD
CCL17 (pg/ml)	`TARC`	V-PLEX Cytokine 30-Plex Kit, MSD
CCL22 (pg/ml)	`MDC`	V-PLEX Cytokine 30-Plex Kit, MSD
CCL26 (pg/ml)	`Eotaxin3`	V-PLEX Cytokine 30-Plex Kit, MSD
CXCL8 (pg/ml)	`IL8`	V-PLEX Cytokine 30-Plex Kit, MSD
CXCL10 (pg/ml)	`IP10`	V-PLEX Cytokine 30-Plex Kit, MSD
MMP-8 total (ng/ml)	`MMP8 Total`	ELISA, R&D Systems DuoSet
MMP-9 activity (arbitrary units)	`Zym`	Gelatin zymography, Invitrogen NuPage
MMP substrate (ng/ml)	`MMPsubstr`	Enzo Life Sciences
Human neutrophil elastase (ng/ml)	`HNE`	ELISA, Mologic
HNE substrate (ng/ml)	`HNEsubstr`	Bachem
Calprotectin (ng/ml)	`Calprotectin`	ELISA, Hycult
Surfactant protein D (ng/ml)	`SPD`	ELISA, R&D Systems DuoSet

Total cell count (× 10^9 cells)	`TotalCellCount`	Microscopy
$CD3^+$ (% of total)	`CD3+live(%)`	Flow cytometry, BD FACSCanto II
$CD14^+$ (% of total)	`CD14+live(%)`	Flow cytometry, BD FACSCanto II
$CD15^+$ (% of total)	`CD15+live(%)`	Flow cytometry, BD FACSCanto II
CD4:CD8 ratio	`CD4/CD8`	Flow cytometry, BD FACSCanto II
$CD4^+$ (% of T cells)	`CD4/CD3(%)`	Flow cytometry, BD FACSCanto II
$CD8^+$ (% of T cells)	`CD8/CD3(%)`	Flow cytometry, BD FACSCanto II
$V\gamma9^+$ (% of T cells)	`g9T/CD3(%)`	Flow cytometry, BD FACSCanto II
$V\delta2^+$ (% of T cells)	`d2T/CD3(%)`	Flow cytometry, BD FACSCanto II

Supplemental Table S4. Proportion of missing values imputed using Multivariate Imputation by Chained Equations (MICE).

**Biomarker**	**Symbol**	**% of missing values**
IL-18 (pg/ml)	`IL18`	3.6
Total cell count (× 10^9 cells)	`TotalCellCount`	4.8
IL-22 (pg/ml)	`IL22`	6.0
MMP substrate (ng/ml)	`MMPsubstr`	6.0
HNE substrate (ng/ml)	`HNEsubstr`	6.0
MMP-9 activity (arbitrary units)	`Zym`	6.0
MMP-8 total (ng/ml)	`MMP8 Total`	6.0
Human neutrophil elastase (ng/ml)	`HNE`	6.0
Calprotectin (ng/ml)	`Calprotectin`	6.0
Surfactant protein D (ng/ml)	`SPD`	6.0
$CD3^+$ (% of total)	`CD3+live(%)`	12.1
$CD14^+$ (% of total)	`CD14+live(%)`	12.1
$CD15^+$ (% of total)	`CD15+live(%)`	12.1
$V\gamma9^+$ (% of T cells)	`g9T/CD3(%)`	12.1
CD4:CD8 ratio	`CD4/CD8`	16.9
$CD4^+$ (% of T cells)	`CD4/CD3(%)`	16.9
$CD8^+$ (% of T cells)	`CD8/CD3(%)`	16.9
$V\delta2^+$ (% of T cells)	`d2T/CD3(%)`	16.9

Supplemental Table S5. Multi-class classification train and validation accuracy of ANN. Accuracy of discriminating between five classes (No growth, Gram-negative, streptococcal, coagulase-negative *Staphylococcus* and other Gram-positive bacteria) achieved by an artificial neural network (ANN) of comparable complexity using 40,000 neurons, which was used as a benchmark value for the TM, reported as mean values of training and validation results, as well as their standard deviation calculated over 5 folds of the stratified *K*-fold cross-validation.

**Dataset**	**Data split**	**ANN (40,000 neurons) performance (%)**			
		Accuracy	Precision	Recall	F1 Score
Full biomarkers dataset (40 soluble, 9 cellular biomarkers)	train	100	100	100	100
	val.	36.62 ± 8.49	29.73 ± 7.93	35.47 ± 5.79	31.32 ± 6.40
Soluble biomarkers (excl. *Zym*, incl. *TotalCellCount*)	train	99.50 ± 1.00	99.53 ± 0.94	99.50 ± 1.00	99.49 ± 1.02
	val.	34.26 ± 9.59	34.73 ± 10.62	32.60 ± 6.94	31.48 ± 8.07

Supplemental Table S6. Train and validation performance of ANNs at different stages of the hierarchical binary classification. The table shows the average performance (accuracy, precision, recall and F1 score) of the ANN of comparable complexity (13,000 neurons) over 5 folds of the stratified *K*-fold cross-validation used at each classification step.

#	**Classification step**	**Data split**	**ANN (13,000 neurons) performance (%)**			
			Accuracy	Precision	Recall	F1 Score
1	No growth *vs* Culture-positive cases	train	100	100	100	100
		val.	81.84 ± 7.46	77.99 ± 11.75	74.55 ± 13.65	72.84 ± 10.99
2	Gram-positive *vs* Gram-negative bacteria	train	100	100	100	100
		val.	69.87 ± 8.86	53.67 ± 16.39	58.61 ± 13.96	55.66 ± 15.03
3	Gram-pos. streptococcal *vs* non-streptococcal bacteria	train	100	100	100	100
		val.	71.56 ± 9.26	72.42 ± 15.13	63.33 ± 8.50	64.01 ± 8.78
4	Coag.-neg. *Staphylococcus* *vs* other Gram-pos. bacteria	train	92.90 ± 4.75	9.331 ± 4.60	92.90 ± 4.75	92.88 ± 4.76
		val.	67.62 ± 10.71	50.33 ± 2.358	55.50 ± 13.08	51.22 ± 17.13

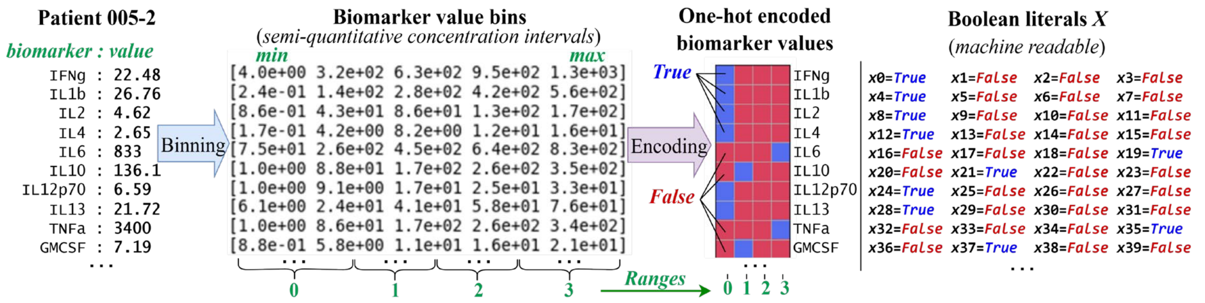

Supplemental Figure S1. Data Booleanisation: binning, encoding and visualisation. The figure shows sample #005-2 as example, from an individual infected with *Proteus vulgaris* (a Gram-negative bacterium). As a result of the Booleanisation, each biomarker value was replaced by a unique binary vector of Boolean features X={x_i} with the same length as the number of used semi-quantitative intervals. In this vector, only one element was set to 1 (*i.e. True;* blue pixel), indicating the presence of the biomarker value in that specific interval, and all other elements were set to 0 (*i.e. False*; red pixel).

```
Clause #0:  x0 ∧ ¬x1 ∧ ¬x2 ∧ ¬x3 ∧ ¬x6 ∧ ¬x7 ∧  x8 ∧ ¬x9 ∧ ¬x10 ∧ ¬x11 ∧ …
Clause #1:  x0 ∧ ¬x1 ∧ ¬x2 ∧ ¬x3 ∧  x4 ∧ ¬x5 ∧ ¬x6 ∧ ¬x7 ∧  x8  ∧ ¬x9  ∧ …
Clause #2:  x0 ∧ ¬x1 ∧ ¬x2 ∧ ¬x3 ∧  x4 ∧ ¬x5 ∧ ¬x6 ∧ ¬x7 ∧  x8  ∧ ¬x9  ∧ …
Clause #3:  x0 ∧ ¬x1 ∧ ¬x2 ∧ ¬x3 ∧ ¬x6 ∧ ¬x7 ∧  x8 ∧ ¬x9 ∧ ¬x10 ∧ ¬x11 ∧ …
Clause #4:  x0 ∧ ¬x1 ∧ ¬x2 ∧ ¬x3 ∧  x4 ∧ ¬x5 ∧ ¬x6 ∧ ¬x7 ∧  x8  ∧ ¬x9  ∧ …
Clause #5: ¬x112 ∧ x114 ∧ ¬x183  ∧ x185 ∧ ¬x192
Clause #6: ¬x21  ∧ ¬x42 ∧ ¬x91   ∧ ¬x117 ∧  x147 ∧ ¬x148   ∧ x157
Clause #7:  x0 ∧ ¬x1 ∧ ¬x2 ∧ ¬x3 ∧  x4   ∧ ¬x5 ∧ ¬x6 ∧ ¬x7 ∧ x8 ∧ ¬x9  ∧ …
Clause #8:  x82  ∧ x114 ∧ x144   ∧ x170
Clause #9: ¬x12  ∧ x156
```

Supplemental Figure S2. Positive clauses (logical rules) recognising Gram-negative bacterial infection. Each clause specifies which Boolean features $X = \{x_i\}$ of a patient sample (see Figure 2) must be *True*, *False* or should be ignored (if the feature is not included in the clause) to support the decision that the patient is infected with Gram-negative bacteria. These logical rules corresponded to clauses inference visualised in Figure 3.

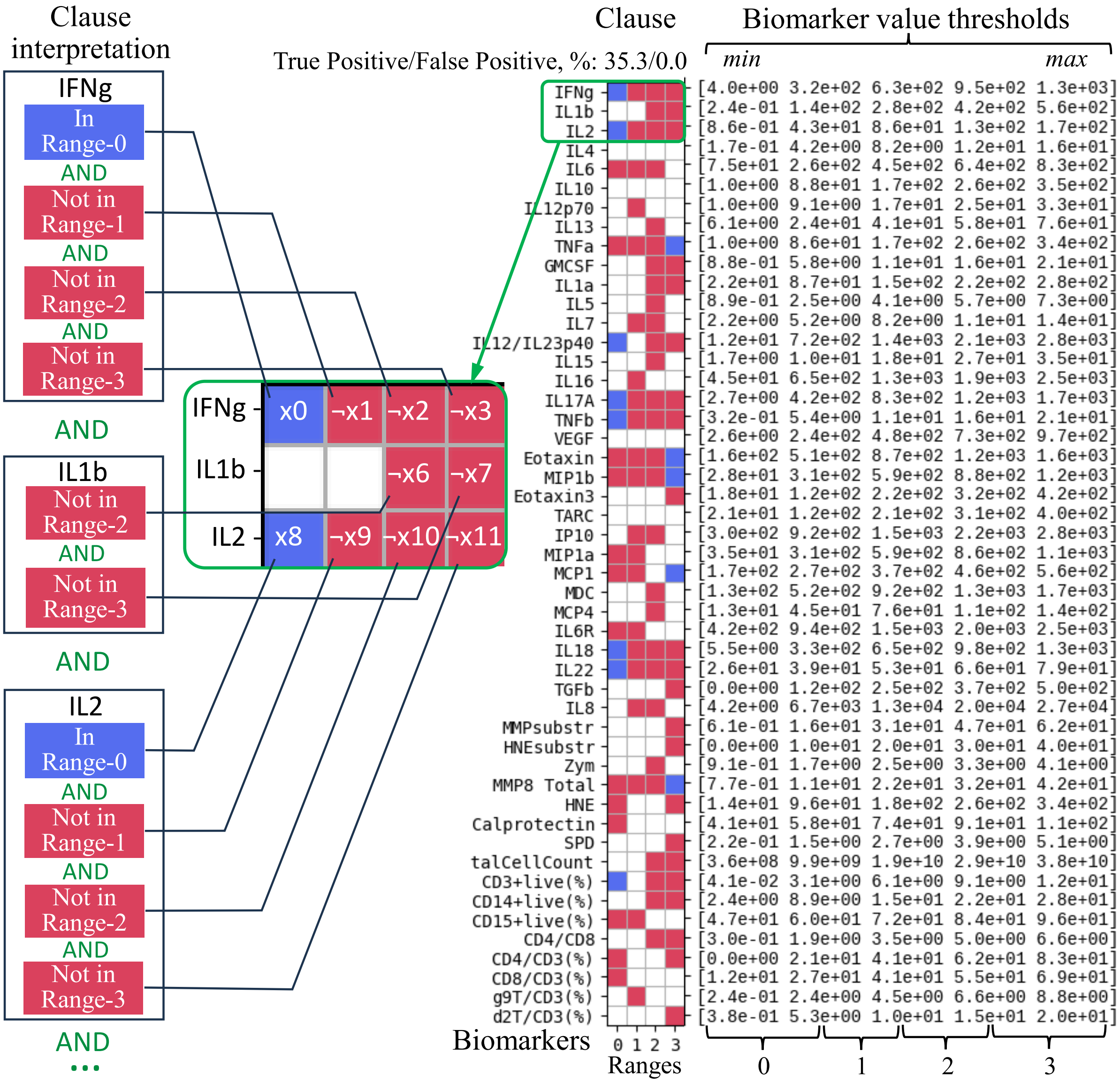

“IF (**IFNg** value IS in Range-0, AND IS NOT in Range-1, AND IS NOT in Range-2, AND IS NOT in Range-3)
AND (**IL1b** value IS NOT in Range-2, AND IS NOT in Range-3)
AND (**IL2** value IS in Range-0, AND IS NOT in Range-1, AND IS NOT in Range-2, AND IS NOT in Range-3)
AND …
THEN the clause supports a hypothesis that a patient is infected with **Gram-negative bacteria**”.

Supplemental Figure S3. Clause visualisation and interpretation. The figure presents an example of a clause supporting decision-making in favour of Gram-negative bacterial infection. A blue pixel means that the corresponding Boolean feature must be *True* (*i.e.* the biomarker value must be within that specific range) for a patient sample to be classified as Gram-negative bacterial infection. Red means that that the corresponding Boolean feature must be *False* (*i.e.* the biomarker value must not be in that range). Finally, white means that the corresponding Boolean feature was excluded from the clause (*i.e.* the biomarker value may or may not be in that range as it does not affect the classification).

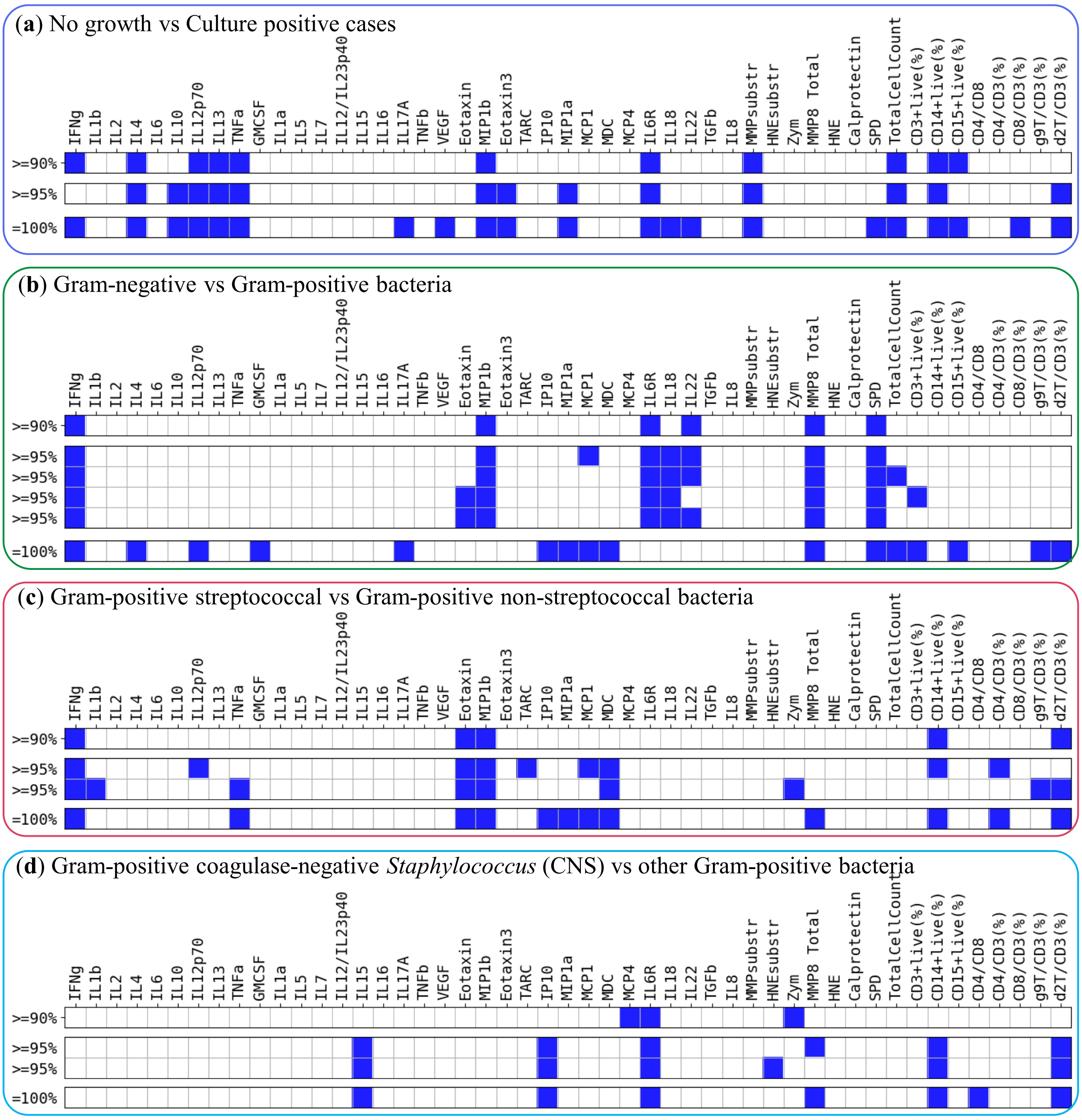

Supplemental Figure S4. Minimised sets of soluble and cellular immune biomarkers for the case where each biomarker value was Booleanised by four semi-quantitative ranges. Figure shows the minimised set of soluble and cellular immune biomarkers needed to make predictions at different classification stages with the target accuracies of 90%, 95% and 100%, respectively.

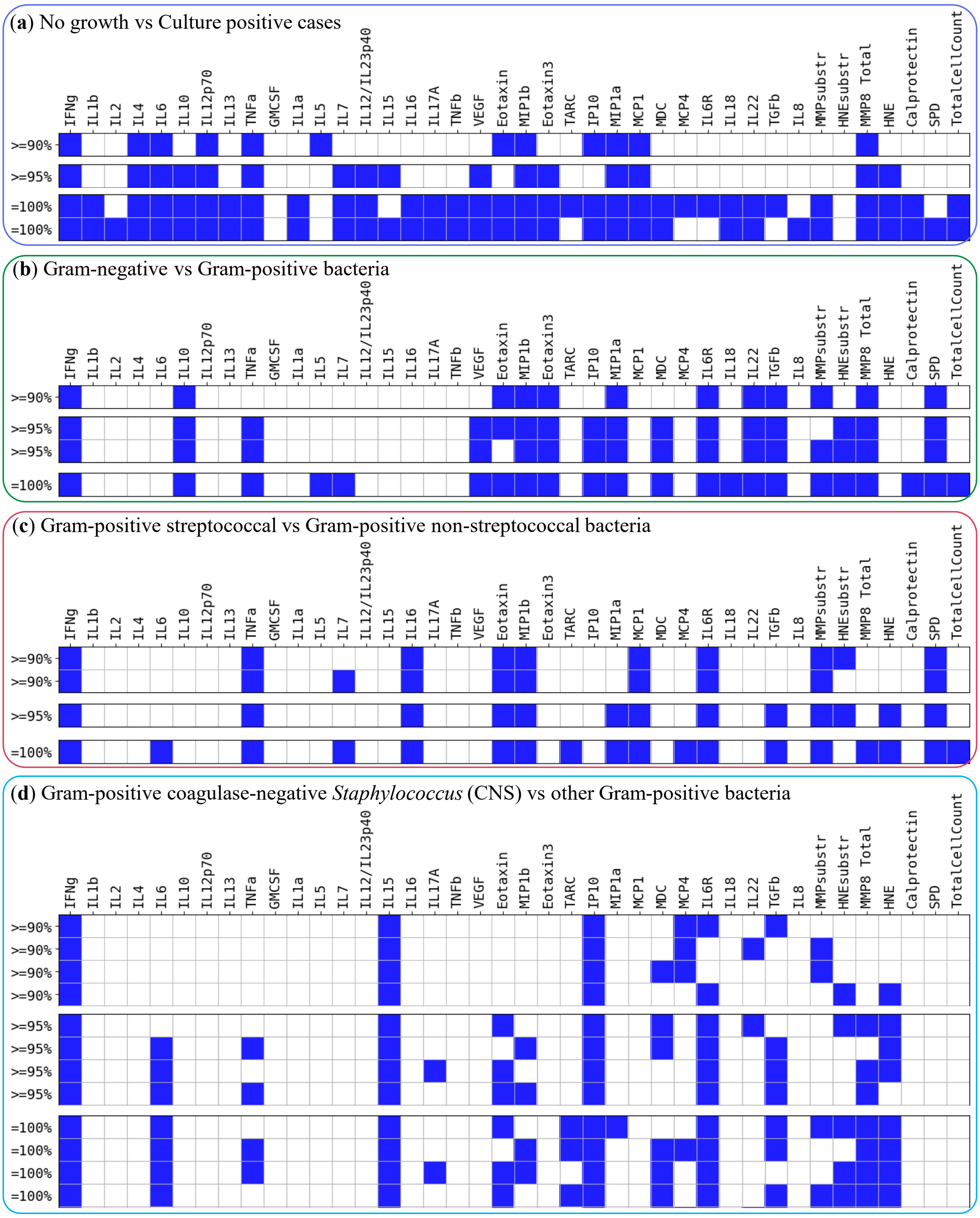

Supplemental Figure S5. Minimised sets of soluble immune biomarkers for the case where each biomarker value was Booleanised by three semi-quantitative ranges. Figure shows the minimised set of soluble biomarkers (excluding *Zym*, including *TotalCellCount*) needed to make predictions at different classification stages with the target accuracies of 90%, 95% and 100%, respectively.

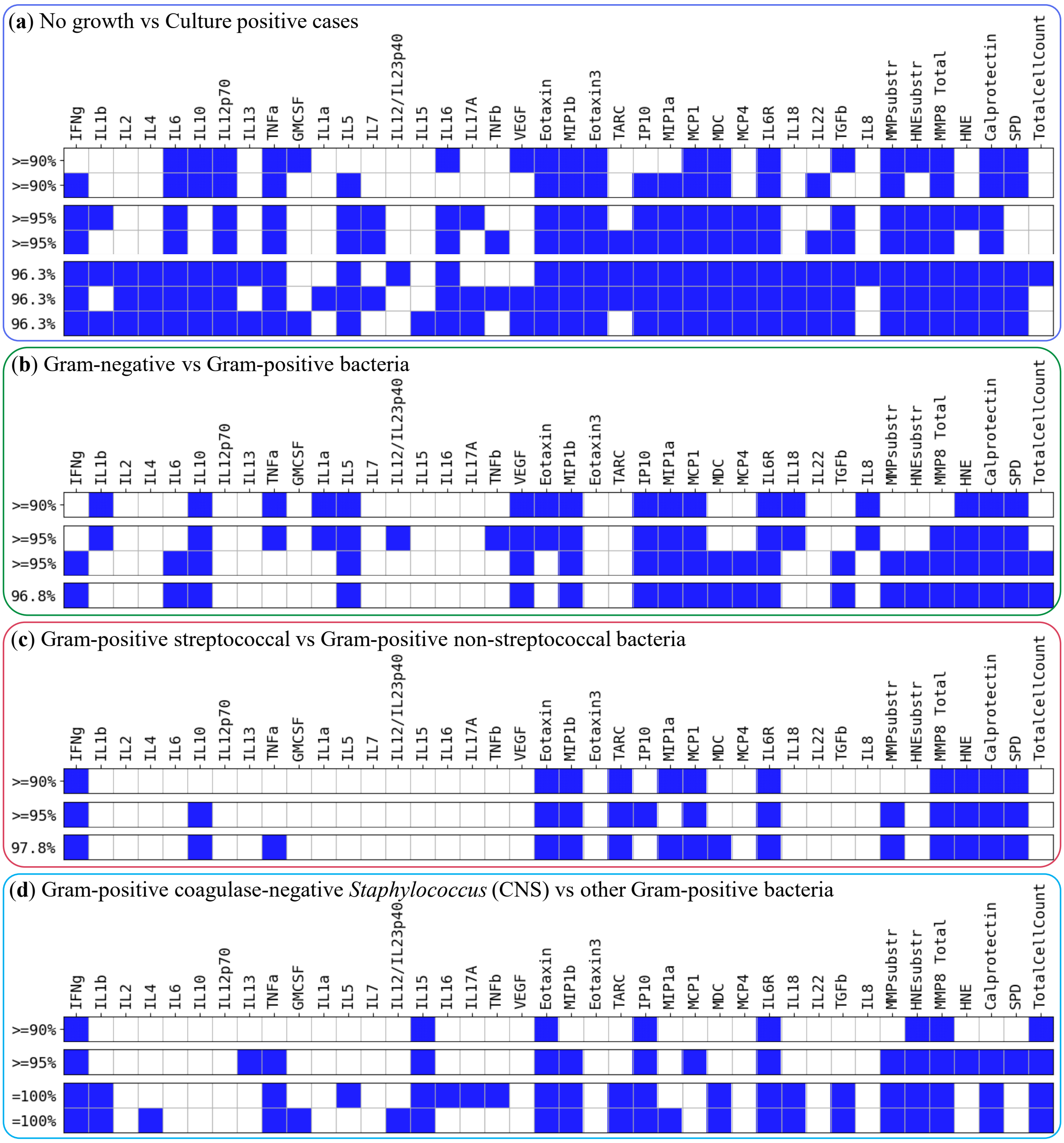

Supplemental Figure S6. Minimised sets of soluble immune biomarkers for the case where each biomarker value was Booleanised by two semi-quantitative ranges. Figure shows the minimised set of soluble biomarkers (excluding *Zym*, including *TotalCellCount*) needed to make predictions at different classification stages with the target accuracies of 90%, 95% and 100%, respectively.
